# Online-Based and Technology-Assisted Psychiatric Education for Trainees: Scoping Review

**DOI:** 10.2196/64773

**Published:** 2025-04-15

**Authors:** Mohd Amiruddin Mohd Kassim, Sidi Muhammad Yusoff Azli Shah, Jane Tze Yn Lim, Tuti Iryani Mohd Daud

**Affiliations:** 1 Department of Psychiatry Faculty of Medicine Universiti Kebangsaan Malaysia Bandar Tun Razak, Kuala Lumpur Malaysia; 2 Department of Psychiatry Hospital Canselor Tuanku Muhriz Bandar Tun Razak, Kuala Lumpur Malaysia; 3 Department of Psychiatry and Psychological Health Faculty of Medicine and Health Sciences Universiti Malaysia Sabah Kota Kinabalu, Sabah Malaysia

**Keywords:** online learning, telepsychiatry, remote learning, virtual, training, education, psychiatry, trainees, residents

## Abstract

**Background:**

The concept of online learning in medical education has been gaining traction, but whether it can accommodate the complexity of higher-level psychiatric training remains uncertain.

**Objective:**

This review aims to identify the various online-based and technology-assisted educational methods used in psychiatric training and to examine the outcomes in terms of trainees’ knowledge, skills, and levels of confidence or preference in using such technologies.

**Methods:**

A comprehensive search was conducted in PubMed, Cochrane, PsycINFO, Scopus, and ERIC to identify relevant literature from 1991 until 2024. Studies in English and those that had English translations were identified. Studies that incorporated or explored the use of online-based or technology-assisted learning as part of psychiatric training in trainees and had outcomes of interest related to changes in the level of knowledge or skills, changes in the level of preference or confidence in using online-based or technology-assisted learning, and feedback of participants were included. Studies were excluded if they were conducted on populations excluding psychiatric trainees or residents, were mainly descriptive of the concept of the intervention without any relevant study outcome, were not in English or did not have English translations, or were review articles.

**Results:**

A total of 82 articles were included in the review. The articles were divided into 3 phases: prior to 2015, 2015 to 2019 (prepandemic), and 2020 onward (postpandemic). Articles mainly originated from Western countries, and there was a significant increase in relevant studies after the pandemic. There were 5 methods identified, namely videoconference, online modules/e-learning, virtual patients, software/applications, and social media. These were applied in various aspects of psychiatric education, such as theory knowledge, skills training, psychotherapy supervision, and information retrieval.

**Conclusions:**

Videoconference-based learning was the most widely implemented approach, followed by online modules and virtual patients. Despite the outcome heterogeneity and small sample sizes in the included studies, the application of such approaches may have utility in terms of knowledge and skills attainment and could be beneficial for the training of future psychiatrists, especially those in underserved low- and middle-income countries.

## Introduction

The incorporation of online-based or technology-assisted methods in medical education is not new. Virtual grand rounds, web-based learning, online journal clubs, and virtual clinical cases and labs are among the many examples of their ubiquitous implementation [1]. The mass adoption of technology-based education is attributed to its numerous perceived advantages, including the ability to transcend geographical boundaries, the presence of learner-centered approaches, the development of students’ self-directed learning skills, and the asynchronous interaction between teachers and students. As education is getting more globalized due to increasing connectivity, these benefits are being increasingly valued [2].

However, questions remain about whether the many advantages of online education are as intuitively apparent and relatable in the field of psychiatry. Traditionally considered as a face-to-face medical discipline, concerns arise regarding the unique interpersonal nature of psychiatry, with its emphasis on empathetic responsiveness toward patients. These concerns are particularly relevant when considering a virtual or simulated patient, and this represents one of the frontier aspects of online education [3]. It is an undeniable fact that appreciating cues from patients is an experiential aspect of knowledge, which is often deemed irreplicable in online sessions.

As technology-based education is increasingly recognized as being noninferior to physical education in undergraduate studies [4], it is imperative to investigate its application in training postgraduate students. The outcome is significant, as it pertains to the production of future psychiatric specialists. This inquiry is especially relevant today, given the radical and drastic transition to technology-based education at all levels due to the recent COVID-19 pandemic [5]. Most medical fraternities are able to integrate online-based and technology-assisted components in their syllabi to enhance the training of trainees or residents without much difficulty. However, acknowledging the distinct nature of psychiatry, which often can be rather ambiguous and subject to nuance, it is important to evaluate the suitability of such an approach to augment the training of future psychiatrists.

Given these considerations, the overarching goal of this study is to systematically map and summarize the existing literature on online-based and technology-assisted psychiatric education for trainees. Specifically, this review aims to identify the various online-based and technology-assisted educational methods used in psychiatric training and to examine the outcome of the aforementioned technologies in terms of trainees’ knowledge, skills, or levels of confidence or preference in using such technologies. We hypothesized that online-based and technology-assisted education can be integrated into psychiatric training to improve trainees’ knowledge, skills, and competency levels.

This review is aimed at psychiatric educators and training directors looking for ways to incorporate technology into their programs, psychiatric trainees who want to understand how online learning fits into their training, and policymakers or accreditation bodies shaping the future of psychiatric education. It is also relevant for researchers and academics interested in digital learning and medical education trends.

## Methods

### Search Strategy

The scoping review was conducted according to the recent methodological framework by Westphaln et al [6], which was derived from the earlier work of Arksey and O'Malley [7]. Five databases (PubMed, PsycINFO, Cochrane, Scopus, and ERIC) were searched from March until June 2024. The keywords applied in PubMed were as follows: ((“resident*”[Title/Abstract] OR “trainee*”[Title/Abstract] OR “postgrad*”[Title/Abstract] OR “graduate*”[Title/Abstract]) AND (“psychiatr*”[Title/Abstract] OR “psychologic* medicine”[Title/Abstract]) AND (“education”[Title/Abstract] OR “training”[Title/Abstract] OR “development”[Title/Abstract] OR “learning”[Title/Abstract] OR “teaching”[Title/Abstract] OR “internship”[Title/Abstract] OR “traineeship”[Title/Abstract] OR “residency”[Title/Abstract] OR “course”[Title/Abstract] OR “lesson”[Title/Abstract] OR “program”[Title/Abstract] OR “programme”[Title/Abstract] OR “class”[Title/Abstract] OR “workshop”[Title/Abstract] OR “module”[Title/Abstract] OR “mooc”[Title/Abstract] OR “academic”[Title/Abstract] OR “clerkship”[Title/Abstract] OR “curriculum”[Title/Abstract]) AND (“on-line”[Title/Abstract] OR “online”[Title/Abstract] OR “digital”[Title/Abstract] OR “virtual”[Title/Abstract] OR “internet-based”[Title/Abstract] OR “internet based”[Title/Abstract] OR “web-based”[Title/Abstract] OR “web based”[Title/Abstract] OR “telepsychiatry”[Title/Abstract] OR “tele-psychiatry”[Title/Abstract] OR “cyber”[Title/Abstract] OR “electronic”[Title/Abstract] OR “e-learning”[Title/Abstract] OR “tele-education”[Title/Abstract] OR “videoconferencing”[Title/Abstract] OR “elearning”[Title/Abstract] OR “distance”[Title/Abstract])).

Different search configurations were used for the databases, and the search strategies are presented in Multimedia Appendix 1.

To ensure completeness, the authors also conducted backward citation searches from key articles and performed searches in Google Scholar to look for grey literature, such as conference proceedings and theses, relevant to the topic. Google Scholar was adopted as it has extensive coverage of academic work and is one of the commonly used search engines for grey literature [8].

### Inclusion and Exclusion Criteria

Acknowledging the emergence of the field and the relatively limited number of studies, the authors made a conscious decision to include a variety of publication types in this review, including original articles, empirical and brief reports, case reports, and short communications. Studies were included in the review if they met the following criteria: (1) incorporated or explored the use of online-based or technology-assisted learning as part of psychiatric training or education; (2) were conducted in populations that included psychiatric trainees or residents; (3) had an outcome of interest related to changes in the level of knowledge or skills or the level of preference or confidence in using online-based or technology-assisted learning, or included feedback of participants on the aforementioned approach (regardless of qualitative or quantitative results); and (4) were written in English or had English translations.

Studies were excluded if they (1) were conducted on populations excluding psychiatric trainees or residents; (2) were mainly descriptive of the concept of the intervention without an evaluation or any relevant study outcome related to the application of online-based or technology-assisted psychiatric education; or (3) were review articles. In addition, studies that only used online questionnaires to conduct pre-post assessments for psychiatric education, which were otherwise not delivered through an online or technology-assisted platform, and studies that primarily assessed the learning needs in online-based or technology-assisted psychiatric education without an evaluation of the intervention itself were also excluded. Studies conducted in languages other than English and those without an English translation were omitted due to limitations in language proficiency and to prevent inaccuracies or misinterpretation of the study findings.

### Data Screening and Extraction

The search results from the 5 databases were exported to Rayyan online reference manager. Duplicates of similar articles detected by Rayyan were screened manually by MAMK and SMYAS to minimize errors in excluding articles. Prior to the title and abstract screening process, both MAMK and SMYAS underwent screening training to promote standardization and to identify possible conflicts. Then, according to the PRISMA (Preferred Reporting Items for Systematic Reviews and Meta-Analyses) approach, the titles and abstracts were screened by MAMK and SMYAS to assess suitability for further examination based on the following predetermined criteria:

- Did the study use online-based or technology-assisted instruments as part of the education approach?
- Was the study focused on psychiatric education or related necessary skills?
- Was the study conducted in samples that included psychiatric trainees or residents?

In this phase, articles were divided into the following categories: accept, maybe, and exclude. Subsequently, the full texts of eligible articles (accept and maybe categories) were retrieved. This was followed by a blinded screening phase during which MAMK and SMYAS independently examined the articles in accordance with the inclusion and exclusion criteria. Any disputes regarding the acceptability of the articles in the title and abstract screening phase and in the full-text screening phase were resolved by an impartial third referee (JTYL or TIMD). Data extraction from all included studies was then conducted, gathering parameters such as author names, study year and country, aims and objectives, interventions applied, and key outcomes of interest. The data were extracted by MAMK and SMYAS to Excel (Microsoft Corp) sheets with predefined data fields.

The quality of the studies was assessed according to the 10-item Medical Education Research Study Quality Instrument (MERSQI) for quantitative studies [9] and the Standards for Reporting Qualitative Research (SRQR) for qualitative studies [10]. Previously, a scoping review adopted the SRQR as a 21-score checklist to assess the quality of included qualitative studies within the review [11].

### Ethical Considerations

This review received ethical approval from the Research Ethics Committee of the National University of Malaysia (JEP-2023-789).

## Results

### Overview of the Included Studies

**Figure 1:**
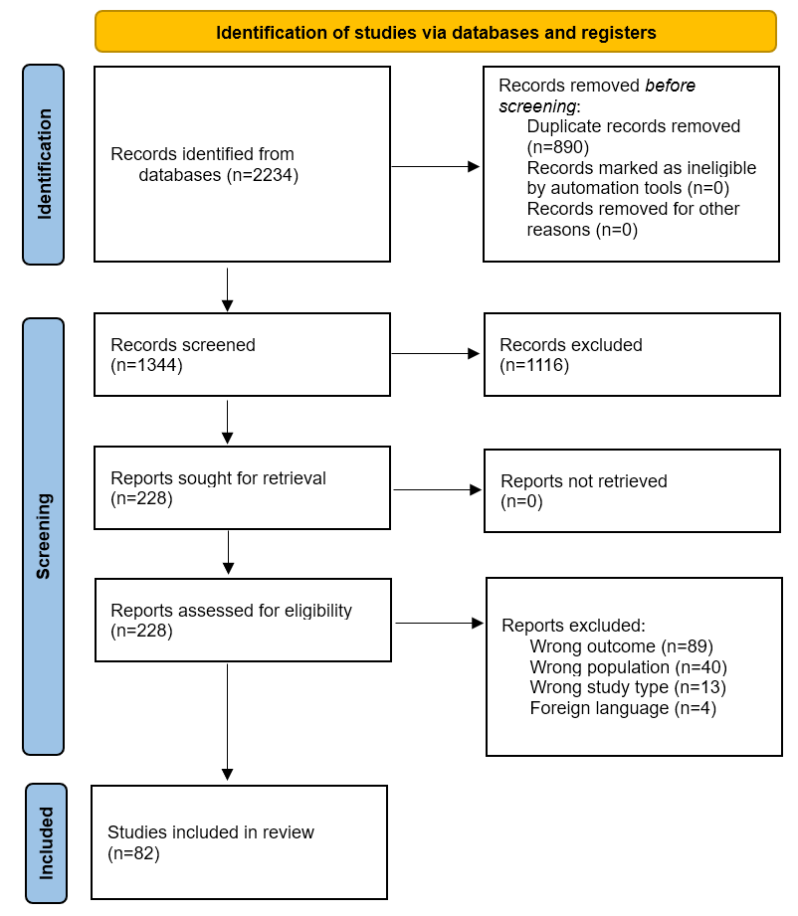
PRISMA (Preferred Reporting Items for Systematic Reviews and Meta-Analyses) flow diagram.

The initial search across the 5 databases yielded a total of 2234 articles, from which 890 duplicates were subsequently removed. After screening the titles and abstracts, 1344 articles were excluded and 228 articles proceeded to full-text screening. Of these, 89 articles were excluded for wrong outcomes (not evaluating the outcome of interest), 40 articles for wrong population (samples excluded psychiatric trainees or residents), 13 articles for wrong study type (review study design), and 4 articles for being in a foreign language. Thus, 82 articles were included in this review (Figure 1).

**Figure 2:**
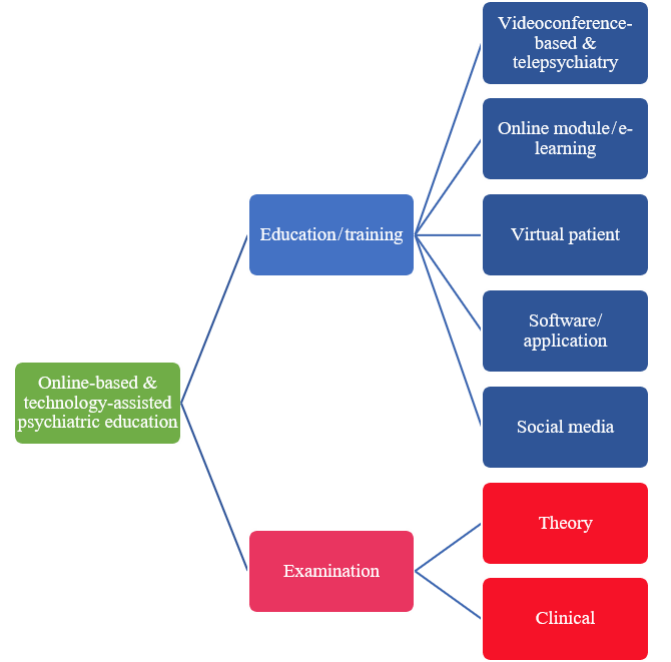
Trend of the implementation of online-based and technology-assisted psychiatric education.

Among the 82 articles included, 2 themes were identified: education and assessment (Figure 2). Under education, the trend could be divided into 5 subthemes, namely online software (or e-learning platform or massive open online course [MOOC]), videoconference (or telepsychiatry), virtual patient (or simulation), software/application, and social media. Under assessment, there were 2 subthemes, namely theory and clinical examination.

### Studies Prior to 2015

**Figure 3:**
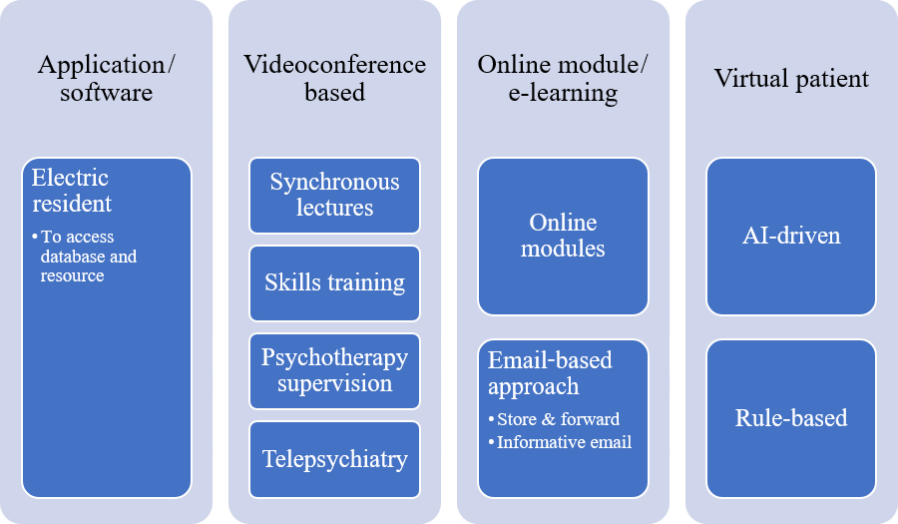
Taxonomy of online-based and technology-assisted learning prior to 2015. AI: artificial intelligence.

A total of 20 articles published before 2015 were included (Table S1 in Multimedia Appendix 2). Specifically, 2 articles were from the 1990s, 9 articles were from the 2000s, and 9 articles were from 2011 to 2014. These articles were mainly from Western countries, such as the United States and Canada. The articles primarily involved telepsychiatry and videoconferencing as tools for education, training, and supervision (n= 10/20, 50%), followed by web-based approaches or e-learning (n= 5/20, 25%), virtual patients (n= 3/20, 15%), and software (n=1/20, 5%). The taxonomy of the methods applied in psychiatric education during this period is presented in Figure 3. Studies mainly had a 1-group, posttest-only study design; 1-group, pretest-posttest study design; and randomized controlled trial design. Out of the 20 articles, 8 had objective measures, such as the change in the level of knowledge before and after the intervention, while the other 12 had subjective measures, such as the level of satisfaction and participant feedback.

Despite the limitation of the internet in earlier years, there have been some attempts to integrate IT as part of training. Within this period, depending on financial capabilities and regions, few types of internet connections were available, for example, dial-up connection (especially in the 1990s), Integrated Service Digital Network (ISDN), and broadband connection. The type of internet connection has influenced the strategy and the experience in the education process. With slower internet, an indirect instructional model or independent study was used, with trainees searching for relevant information to enhance their knowledge in particular topics or cases that they had been consulted on. One of the earliest studies highlighted the use of computers and the internet for accessing MEDLINE to assist residents with their consultations, checking drug interactions, and reviewing literature pertinent to cases that they were consulted on [12]. In a survey, printed materials were preferred when learning something new, but digital media or online resources were preferred when revising or searching for resources during patient care [13].

As seen in other fields of medicine, the advancement of relevant infrastructure enabled faster internet connectivity, which allowed widespread and accessible knowledge sharing through videoconference-based seminars, and this is also applicable in the field of psychiatry. This has allowed direct instructional models through approaches such as online lectures and seminars. However, these approaches were met with mixed feedback. One study in the United States in 2004 highlighted that satisfaction with videoconference-based lecturers was contingent on the internet speed, with trainees or residents in centers having a slower internet speed reporting less satisfaction with the lecture experience and the overall sound quality [14]. Another study in Australia in 2008 reported higher preference among participants to attend seminars from remote sites, and most participants felt that the videoconference-based seminars were beneficial for their practice [15]. Meanwhile, in a study in South Africa by Chipps et al [16], while videoconferencing was perceived as an excellent education tool by half of the psychiatric registrars, only 39% of them felt that it was as effective as face-to-face teaching. This led to decreased interest in further videoconference-based training. Additionally, another randomized controlled trial in Iran in 2014, which aimed to compare the effectiveness of face-to-face communication skills training sessions against distant learning in improving empathy, found that the level of empathy was significantly increased in the attending group but not in the distant learning group [17].

One study in Norway in 1998 explored the use of videoconferencing technology in terms of psychotherapy supervision [18]. The psychiatric trainees conducted face-to-face psychotherapy sessions with their patients and later had alternating face-to-face and online psychotherapy supervisions with their supervisors. Through semistructured interviews after the completion of the psychotherapy session, it was noted that while the reduced nonverbal cues were an issue, the limitations of the videoconferencing supervision paradoxically had some positive effects among the trainees in terms of the supervision process, such as verbalization and structure. The positive effects were also contributed by the ease of logistics and by having a neutral space separate from the supervisor’s office.

As an extension to videoconference use, telepsychiatry serves as a valuable tool to expand the reach of psychiatric services. As such, it has been incorporated as part of training for psychiatric trainees or residents. In terms of supervision, most of the studies included direct, side-by-side supervision by attendings for assessing patients [19-22]. On the other hand, 1 study adopted a different approach, with attending psychiatrists sitting in with residents during their first session to help familiarize them with conducting treatment via telepsychiatry, and in later sessions, the involvement of supervising attendings was on an “as needed basis” [23]. Most feedback by trainees on telepsychiatry programs indicated that telepsychiatry enhanced their skills and knowledge, with majority of trainees stating that it was interesting and enhanced their training. However, some trainees mentioned technical issues with this approach and the difficulty in assessing the influence on patients.

On the other hand, improved access to the internet has expanded the utility of asynchronous learning methods. In earlier years, a web-based email approach was applied to promote exposure or learning about stigma education [24] and child and psychiatry cases [25] through approaches such as the “store and forward” concept. However, in Western countries where technology was more advanced compared to the rest of the world at that point of time, e-learning materials were typically in the form of slides of didactic content with recorded audios and videos. This delivery method was used in learning evidence-based medicine [26] and to improve electrocardiogram reading skills [27]. A study by Garside et al [28] in 2009 managed to introduce direct and interactive instruction strategies to learn about how to fill Form 1 of the Mental Health Act. This was achieved by integrating slides of relevant materials regarding Form 1 and the laws related to it, together with interactive Flash animations and practice cases, using questions, and there was immediate expert feedback for each question. Throughout these studies, there were statistically significant improvements in the levels of knowledge and skills of the trainees, suggesting the potential of such an approach to augment the training of psychiatric trainees.

Interest in a virtual patient as an education tool for psychiatric training emerged in the 2000s and 2010s, and facilitated more immersive learning. Within this period, 2 types of virtual patients were studied: artificial intelligence (AI)-driven virtual patients and rule-based virtual patients. Kenny et al [29] and Pataki et al [30] described the use of an AI-driven virtual patient to simulate an adolescent patient with posttraumatic stress disorder (PTSD) (“Justina”). The virtual patient was developed according to the criteria of PTSD based on the Diagnostic and Statistical Manual of Mental Disorders (DSM) [31] and involved technologies such as voice recognition, response selection, behavior generation, and a visual graphics engine. “Justina” received good feedback from residents who mentioned that the experience they had from assessing the virtual patient closely matched their actual experience, but there were times when the virtual patient was not able to understand the questions from the residents. In another study, the rule-based virtual patient concept was applied to assess the doctor’s competence in obtaining informed consent before prescribing antipsychotics in a simulated patient with psychosis [32]. A Flash-based video was shown to introduce the clinical scenario, followed by a series of menu options from which they could choose their next action. After completion of the scenario, the program provided a feedback screen regarding the appropriateness of their actions, as well as a link to relevant resources to improve their knowledge. There were statistically significant improvements in all items of the Confidence Scale (pretest to posttest).

### Studies From 2015 to 2019 (Before the COVID-19 Pandemic)

**Figure 4:**
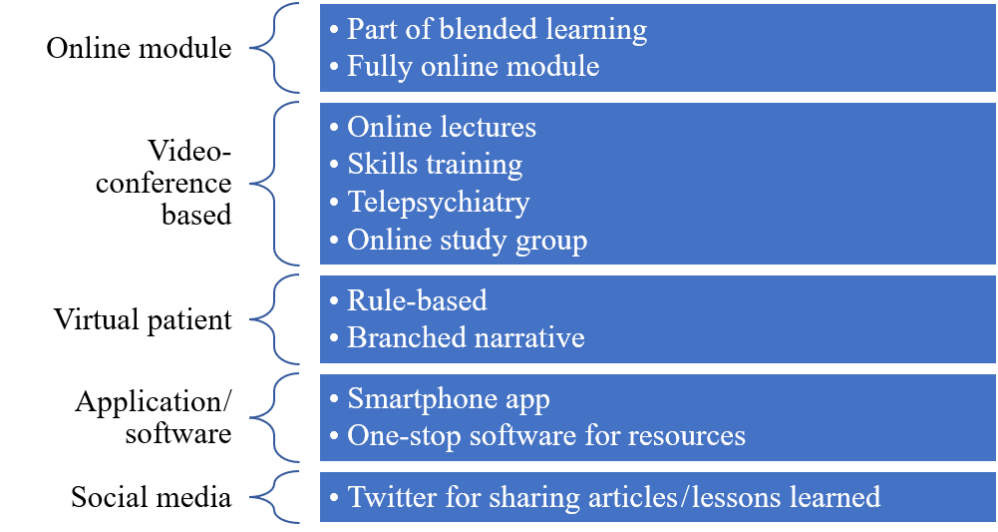
Taxonomy of online-based and technology-assisted learning in 2015-2019.

During the period from 2015 to 2019, there was an acceleration of internet-based or technology-assisted education in psychiatry, with 21 studies identified over 5 years (Table S2 in Multimedia Appendix 2). Western countries, particularly the United States and Canada, were at the forefront of these initiatives. Majority of the studies involved videoconference-based learning (n= 7/21, 33.3%), followed by use of online modules or e-learning platforms (n= 5/21, 23.8%), development of software or applications (n= 3/21, 14.3%), adoption of virtual patient approaches (n= 3/21, 14.3%), and use of social media as a learning tool (n= 1/21, 4.8%) (Figure 4). The most common study design was a 1-group, pretest-posttest design, followed by a 1-group, posttest-only design. There was only 1 randomized controlled trial and 1 crossover intervention study. Studies mainly applied subjective measures to assess the outcome of interest, and there was an increasing trend of objective measures in 9 studies, mainly to assess changes in knowledge.

There was more organized and comprehensive use of online resources, with UpToDate, PubMed, Wikipedia, and e-journals being popular among trainees [33]. However, some residents cited insufficient time, insufficient faculty guidance, and lack of resources specific to psychiatry as barriers to using online resources in their practice. Moreover, 86% of respondents felt that there is a need for more psychiatry-specific online resources, and 79% believed that online resources should be more visual and interactive. In another survey, some trainees preferred to read or take notes on paper for academic purposes [34]. However, they still preferred on-screen reading for checking medication dosing and information.

As accessibility to the internet improved with wider coverage and greater stability, there was an increased effort to conduct online modules or e-learning to complement the traditional methods of psychiatric learning, at least for delivering theoretical aspects. For example, Hickey et al [35] conducted a blended course of traditional lectures, online modules, and videotape reviews in psychotherapy education. The authors developed 2 modules on Davanloo Intensive Short-term Dynamic Psychotherapy (IS-DTP), which consisted of PowerPoint presentations, videos, and pre- and posttests. In a crossover intervention design, residents were divided into 2 groups, with each group receiving an online module and a face-to-face lecture but in a different order for 2 topics. It was found that there was a statistically significant improvement in knowledge acquisition in both the online module group and face-to-face lecture group, and there was no significant difference in comparison between both groups regardless of how the topics were delivered.

Three studies described blended learning but with a different model compared to the earlier study [36-38]. For example, a bookend blended learning model was adopted, in which there was self-paced virtual learning via online modules in the beginning, followed by synchronous in-person interactive discussion, and the approach ended with case supervision or reflection. This approach was used in learning PTSD [36] and in an integrative psychiatry curriculum [37], and residents appreciated the convenience and suitability to augment their training owing to 2 main factors. The first factor was that the self-paced online modules allowed for more time for personalized training and to process materials, and the second factor was the ability to access quality information linked directly to published sources. In another variation of blended learning, another study applied the flipped classroom model, in which the required reading was provided earlier, and then, participants engaged in interactive asynchronous discussion [38]. Interestingly, less than half of the participants indicated improvement in their knowledge, while the rest felt no difference, and a small number of participants felt that the approach had a negative impact on their knowledge, indicating the need to re-evaluate the suitability of the asynchronous flipped classroom approach for psychiatric training.

Fully online courses or modules have been continuously adapted to different aspects of psychiatric training. Brief online courses or modules were employed in rather specific topics under psychiatric-related theoretical knowledge, such as catatonia [39], tobacco use disorder [40], and substance use disorder [41]. Certain modules can be completed in 10 minutes, and brief modules often include slide presentations and relevant videos (recorded webinars, patient interviews, or demonstrations of symptoms and signs), which trainees can complete at their own pace. Despite the brevity of the approach, there were significant increases in knowledge [39,40] or improved levels of stigma [41] between pre- and posttests, with some of the improvements being retained at the 3-month or 6-month follow-up, suggesting the utility of such an approach.

Videoconference-based discussions or webinars remain useful tools in psychiatric education. Few studies used videoconference platforms to deliver online lectures and online case discussions tailored for aspects of psychiatric training, such as career development activities [42] and continuous education in conflict zones [43]. Videoconference platforms have also been used for skills training. Puspitasari et al [44] conducted a study on online behavioral activation training over 4 weeks with the aim to compare trainer-led online training and self-paced online training. There was a significant increase in behavioral activation skill assessment total scores in both groups, and there were significant between-group differences favoring the trainer-led online group at both the posttraining assessment and 3-month follow-up. Although direct instructional strategies were applied in both groups and both approaches were conducted online, the interactive component of the session in the trainer-led group caused a significant difference in the improvement of the skills of the trainees.

Building on the earlier work of Pignatiello et al [21] and Volpe et al [22], a study by Teshima et al [45] focused on feedback from over 300 residents in telelink telepsychiatry training in Canada. The trainees appreciated the opportunity to learn about different approaches to interviewing clients. Although the residents found the technology a “bit unnatural” at the beginning of the session and realized that it was “challenging to interview [patients] at a distance,” they were still able to obtain nonverbal cues, which were important to understand their patients. Almost all participants agreed that the telepsychiatry experience was interesting, while 97% agreed with the statement “the experience helped me understand more about providing psychiatric services to underserved areas.” As the telelink program was an established and mandatory program for psychiatric trainees in University of Toronto, this study had one of the largest sample sizes in comparison to other studies, enhancing its reliability despite its single study site.

Davidson and Evans [46] illustrated how a videoconference platform was employed for an online study group as an augmentation tool in preparing for the Royal Australian and New Zealand College of Psychiatrist (RANZCP) objective structured clinical examination (OSCE). Four New Zealand trainees used Google Hangout (now known as Google Meet) for their online OSCE practice, with the exam questions based on online past papers on the RANZCP website [46]. The members rotated their role to be a candidate, examiner, or role-player, adding to the experiential learning. The 4 trainees passed their OSCE and acknowledged the benefit of a virtual study group in enhancing their preparation.

As demonstrated by earlier studies, the concept of a rule-based virtual patient was proven useful in learning PTSD and significantly improved the knowledge and confidence level of residents in managing PTSD [47,48]. In another variation, Wilkening et al [49] employed a branched-narrative virtual patient for advanced psychopharmacology sessions, in which residents were presented with a challenge and given choices, and the consequences would depend on the choice selected. The integration of the virtual patient concept led to statistically significant improvements in knowledge levels in advanced psychopharmacology, supporting the efficacy of the virtual patient concept as part of psychiatric education.

Abundance of reliable resources is a boon to evidence-based medicine; however, due to the hectic nature of clinicians, a quick decision must be made quite often. Thus, few software programs were developed to act as a 1-stop center for reference to help expedite and guide their practice. Adeponle et al [50] developed the Psychiatry Toolkit, which allowed direct, immediate, and full access (institutional login) to desired journals, articles, and relevant databases, including PubMed, PsycINFO, and UpToDate [50]. Another study by Dirlam et al [51] described Mental Health EMR Tools, which is a large database that allows residents to access prevetted, curated, and continuously updated information to help with their clinical practice. In addition, acknowledging the potential of a smartphone as an educational tool, Zhang et al [52] developed the Delirium University Health Network Application as a tool for delirium education. It was initially developed as an online application and was later piloted as a smartphone app via the Android Play Store. The app included many important contents related to delirium, such as the DSM-5 diagnostic criteria for delirium, common causes of delirium, pharmacological and nonpharmacological interventions for delirium, and objective assessment questionnaires (eg, Confusion Assessment Method [CAM]). Overall, Mental Health EMR Tools and the delirium smartphone app received good feedback from users, who appreciated the convenience of getting information from a consolidated source. On the other hand, while the Psychiatry Toolkit helped residents to look for answers to their clinical questions, the adoption rate of the toolkit among respondents was relatively low at 47% [50].

An interesting study described an innovative approach to adjunct psychiatric education using social media. Walsh et al [53] described the use of Twitter to disseminate education resources considered helpful in training. Under Twitter account @PhippsPsych, residents took turns to post tweets or retweet contents, such as take-home points from psychiatry grand rounds, links to journal articles, and references to psychiatry in current events. While the study had a rather small sample size with 49 residents, there was a significant increase in the proportion of participants using Twitter for medical education from 8.2% to 28.6%. However, residents’ ratings regarding the usefulness of social media in medical education did not change from pre- to postsurvey, and corroborated by the fact that 60% of residents reported that the knowledge gained from following the account had no impact on their clinical practice, 37.2% reported a minimal or average impact and only 2.8% reported a great impact.

### Studies From 2020 Onward (After the COVID-19 Pandemic)

**Figure 5:**
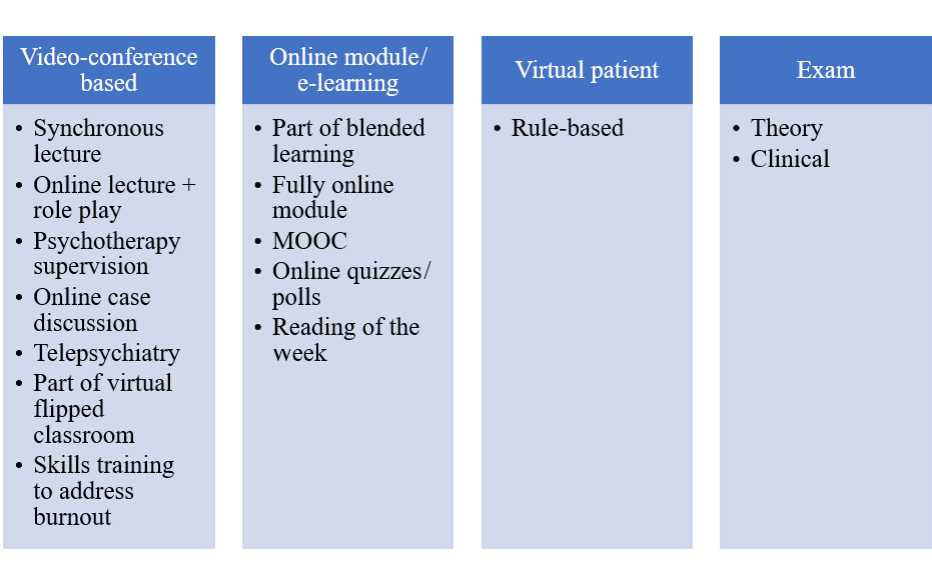
Taxonomy of online-based and technology-assisted learning in 2020-2024. MOOC: massive open online course.

The number of articles on online-based or technology-assisted learning in psychiatry education for psychiatric residents or trainees saw a significant spike during and after the COVID-19 pandemic. There were 41 relevant articles from 2020 until June 2024 (Table S3 in Multimedia Appendix 2). While the United States, Canada, and the United Kingdom had the highest number of studies, there was also notable involvement from Global South countries, such as Malaysia, Thailand, Pakistan, India, and Tunisia. Majority of the articles involved the application of videoconference-based learning or webinar concepts (n= 24/41, 58.5%) and online modules (n= 8/41, 19.5%) (Figure 5). Studies mainly had subjective measures as outcomes and had a 1-group posttest-only study design. There were 11 studies with a 1-group pretest-posttest design and 1 study with a nonequivalent group posttest-only design.

Transition to videoconference-based learning and webinars was necessary for the continuation of training and education during the COVID-19 pandemic, including for psychiatric residents. There were few variations in how videoconference-based learning was applied. In some articles, it was rather straightforward with synchronous online lectures through the videoconference platform, and some of the lectures were then followed by virtual group discussions or brainstorming sessions in the breakout rooms of the platform to make it more interactive. This approach was commonly used in the theoretical aspects of psychiatric training, such as in alcohol use disorder [54], fundamentals of remote psychotherapy [55], research in psychiatry [56], digital psychiatry [57], complex child and adolescent cases [58], biostatistics and methodology courses [59], and journal clubs [60].

In another variation, the online lecture was paired with virtual role-play or simulation sessions. With the added experiential learning component, residents were able to apply their knowledge accordingly in case scenarios. In a study by Blamey et al [61], psychiatric trainees attended a 2-hour virtual lecture on the necessary skills for their on-call work, and then, the trainees participated in a series of 2-hour simulated on-call shifts once a week for 10 weeks, covering 10 common scenarios for psychiatric on-call work. Acknowledging the importance of understanding the complexities of health systems in delivering effective and safe patient care, Li et al [62] developed an online curriculum for core competencies in health systems science. The residents underwent 10-minute virtual didactics prior to the virtual simulation of case scenarios using the Zoom platform.

Looking at the outcomes, trainees or residents perceived a high level of satisfaction with the program and its online delivery, as well as an increase in confidence in skills and perceived learning gains [56,57,60]. Among the studies, 1 study had an objective outcome, in which significant improvements in the knowledge level regarding alcohol use were noted among residents after the videoconference-based lecture and recorded training video session, indicating the potential efficacy of such a program [54]. The virtual role-play concept was especially credited to be a useful technique to enhance the interactive learning of residents [61]; however, the virtual format can be awkward owing to the need for turn-taking, which in turn affects the interactivity, especially when there are few residents involved concurrently in a single case scenario [62].

As demonstrated by Gammon et al [18], a videoconference platform may be used for psychotherapy supervision. Due to the COVID-19 pandemic, this was of value, and there were 2 studies involving psychiatric trainees receiving psychotherapy supervision virtually. There were concerns regarding the loss of nonverbal cues or subtleties of communication during remote supervision, in addition to the “Zoom fatigue” phenomenon, all of which influenced residents to favor face-to-face supervision more than remote supervision [63]. However, the flexibility of remote supervision and the option to allow residents to attend the supervision even when they were busy with their ward work or were on-call were certainly advantageous, and the quality of psychotherapy skills attainment based on subjective assessments by supervisors was not significantly different between remote supervision and traditional face-to-face supervision [63,64]. In fact, as reported by Famina et al [65], supervising attendings noted that the quality of psychiatric care was not different between remote sessions and in-person sessions, and there was not much difference in terms of the ability to empathize and to interpret nonverbal cues.

With regard to telepsychiatry, the authors of an article mentioned their experience of a sudden unprecedented change to their service, which involved a transition to telepsychiatry due to the COVID-19 pandemic [66]. The service initially involved phone consultation, and residents and attendings subsequently switched to video consultation after obtaining approval to use a video platform [66]. While both trainees and attendings strongly agreed that the change to virtual care was necessary, the attendings felt that trainee supervision and training worsened during the pandemic. The trainees also felt less comfortable conducting virtual care and less confident in their assessments to the extent that they found video consultations “frustrating,” especially when attempting to interview patients who had difficulty engaging in virtual interactions (eg, those with delirium, neurocognitive disorders, or mania) [66]. This experience was echoed in a survey by Cruz et al [67], which showed that the top 5 concerns shared by residents and the faculty about telepsychiatry were the inability to perform a physical exam, poor internet connection, unknown liability risks related to telepsychiatry, certain cultures being less accepting, and nonverbal cues being missed.

Contrary to that experience, the authors of another study described their telepsychiatry experience in a rather positive note. Because of a rapid shift, telepsychiatry sessions were still following the prior model of in-person direct supervision involving attendings and residents, and both had to don a mask while conducting the telepsychiatry sessions, which affected the voice projection and the ability of the patient to hear the treatment plan [68]. Subsequently, with further understanding of the videoconference platform, they were able to continue direct supervision with slight modification as attendings joined the session from their private offices, allowing residents and attendings to remain mask-free and improving the audio for patients. Majority of the residents felt that telepsychiatry had positively impacted their clinical education experience, and it was significantly associated with comfort with practicing telepsychiatry in the future [68].

The flipped classroom model, which is a type of blended learning, reversed the settings, with direct instructions to be provided at home and learning activities involving higher order thinking to be done at school. COVID-19 restrictions necessitated the change to a fully virtual flipped classroom, and psychiatric training was not an exception. A study from Pakistan described a program using the flipped classroom model for an online trauma curriculum [69]. Under this program, reading materials and videos were provided to trainees earlier, and then, the trainees participated in virtual brainstorming, role-play, and case-based discussions. On a larger scale, the Metis didactic courses for psychiatric residents in Sweden (the pedagogical model has been in line with the flipped classroom concept from its inception in 2007) were switched to a digital format to ensure continuation of learning [70]. Each course consists of 3 phases: distance-based self-study, classroom-based meeting days for lectures and supervision, and distance-based examination. The second phase was subsequently transitioned to an online classroom for the same activities. While the fully online flipped classroom concept improved the level of knowledge and skills of psychiatric trainees, some residents preferred to return to face-to-face learning [69,70]. Interestingly, female participants and those aged younger than 50 years were more inclined to continue with online-based course meetings [70].

There was a growing issue of burnout among psychiatric trainees due to the pandemic and its consequences. As such, training programs included skills training as a necessary curriculum component to address burnout, and these were delivered virtually using videoconference platforms, such as the virtual Balint group [71,72], Mind-Body skills program [73], brief mindfulness-based cognitive behavioral therapy (CBT)-informed virtual well-being program [74], and virtual medical improvement program [75]. An interesting example was the virtual Balint group, an initiative that provides a cathartic space and helps to improve morale. With the idea to improve the understanding of patients’ problems rather than finding solutions, residents were encouraged to participate with the camera on and the mic on mute when someone was presenting, and the presenter was free to express their experience of doctor-patient interactions, with the guarantee of nonjudgment and confidentiality [72]. During the discussion, the hands-up function of the Zoom platform was used when someone wanted to speak in order to control flow and avoid interruption. Participants were very positive of the virtual Balint group, with trainees feeling well supported by this initiative. However, many participants preferred face-to-face sessions but nevertheless would choose an online session over no session at all. In another study, the virtual Balint group was credited for promoting a sense of connectedness among peers and providing freedom to speak without needing to censor themselves. However, the virtual nature of the Balint group itself led some participants to feel an abrupt ending to the session, which does not occur in face-to-face sessions [71].

In terms of online modules, the most common design was problem-focused case vignettes, alongside interactive presentation and audiovisual content. Some of the modules also had tests at the end. This design was adopted in few of the studies to cover various aspects of psychiatry training, such as forensic psychiatry [76], catatonia [77], neuropsychiatry [78], tobacco use disorder [79], cultural sensitivity [80], and antiracism intervention [81]. A study by Owais et al [82] used the same online module concept in a blended learning approach for an electroconvulsive therapy curriculum, together with didactic seminars and hands-on clinical management. By combining indirect and direct instruction strategies, there was significant improvement in terms of knowledge attainment after the modules (smaller sample sizes) [76-78,82], and generally, the modules had high satisfaction levels reported by residents [80,81].

While most modules were confined to certain training institutions or regions, 1 article described a larger-scale MOOC to augment psychiatric training. Gargot et al [83] described the First European Psychiatric Association MOOC on CBT, which lasted for a month. With a focus on the theoretical aspects of CBT through recorded lectures, presentations, online forums, and online examinations, the self-paced MOOC had large participation, with 7116 participants enrolling from at least 49 countries. Although the eventual completion rate was 26%, a large number of participants (n=1828) completed the MOOC and the average score for the tests increased steadily from 21.4 out of 25 in the first week to 23.13 out of 25 in the final week, indicating the potential of the MOOC to fill the training gap.

In another study, a website was developed as an innovative, free psychiatry Continuing Professional Development (CPD) resource for Canadian psychiatrists and residents. Referred to as Reading of the Week, this website summarizes the latest psychiatric literature, provides expert commentaries, and promotes discussions on social media platforms [84]. The innovations in psychiatric education as described in these articles received good feedback from trainees. For the Reading of the Week initiative, in which the survey evaluation was based on the 6-level evaluation framework by Moore et al [85], positive feedback and satisfaction were reported by participants across the 6 levels, including knowledge outcomes (level 3), behavior outcomes (levels 4 and 5), and practice outcomes (level 6) [84].

The pandemic also forced the examination process to be performed in a digital format. Generally, examinations in psychiatric training can be divided into theory examinations and clinical examinations (including OSCE). The Royal College of Psychiatrists conducted Member of the Royal College of Psychiatrists theory examinations via a digital platform using a combination of AI and in-person online proctoring [86]. Multiple choice questions were directly assessed, but for questions involving very short answers, smart algorithms were developed to recognize versions of correct answers, and answers that were nonexact matches were reviewed by a designated examiner. On the other hand, for the assessment of clinical psychiatry skills, there was mixed feedback from both examiners and trainees. Depending on the format of the clinical examination, examiners generally manned the stations or the breakout rooms. Integrating videoconference technology for the purpose of clinical examinations had inherent issues, such as connectivity problems, a sense of disconnect, lack of a framework to mentally reset, difficulty in building rapport, and an inadequate capacity to assess clinical skills [87]. However, some residents believed that online assessments were convenient for both participants and patients, reducing anxiety by being in a familiar environment and improving patient access [88]. Some of the candidates even stated that virtual communication was nearly as good as face-to-face communication and online examination was “better than expected” [89].

There is a paucity of studies comparing online training or learning with face-to-face or in-person training. In a quasiexperimental study in Germany to explore whether the satisfaction of online CBT training is noninferior to that of in-person CBT training, the 2 study groups had the same theoretical CBT content, a similar duration of training, comparable audiences, and an identical trainer [90]. The online training was conducted according to the inverted-classroom concept, with participants being required to watch recorded video lectures on the Moodle platform and then have a Zoom discussion at a fixed time for 6 to 7 sessions. It was found that evaluations of the online training group were noninferior to those of the in-person group in terms of information content, didactic presentation, assessment of the trainer as a suitable role-model, working atmosphere, own commitment, and practical relevance, suggesting that the delivery of CBT knowledge through an online platform may be sufficient.

In another study, Hewson et al [91] described a rather indirect comparison between face-to-face basic psychiatry skills simulation training and synchronous online training. The transition to online training via Zoom was due to evolving COVID-19 restrictions at that time and was not in the initial plan. In subgroup analyses, the face-to-face group showed statistically significant improvements in confidence across all psychiatry skills tested, whereas the online group showed significant improvements in confidence in all but 2 skills, namely psychiatric risk assessment and assessment of physical health problems in elderly patients with cognitive impairment. However, the face-to-face group included foundation doctors (junior doctors) and the online group included psychiatry and general practitioner trainees, suggesting that the lack of a significant improvement in confidence in those 2 skills could be related to a higher baseline self-confidence level prior to the simulation training.

A rule-based virtual patient appears to be a mainstay model of a virtual patient in psychiatric education. Rakofsky et al [92] developed a virtual patient-based assessment simulator as a tool to assess the proficiency of residents regarding psychopharmacological knowledge and practice. Combining virtual human avatars, AI, and an advanced pedagogical design, it allows for a realistic interaction, including live voice communication. According to the rule-based virtual patient concept, residents had choices of questions and answers to choose from, and they were given immediate feedback on all their choices alongside the rationale. Looking at the performance of the residents, the mean total score of the simulator by class correlated significantly with the mean score of the somatic therapies subscale of the Psychiatry Residency in Training Exam (PRITE), suggesting construct validity of the virtual patient simulator.

In a survey assessing residents’ perceptions of the pandemic’s impact on their didactic experience and training preferences, it was found that trainees appreciated several positive aspects of virtual didactics, such as being easy to attend and being engaging, and they were able to invite guest speakers from other institutions easily [93]. However, some negative experiences were also reported, including the “Zoom fatigue” phenomenon and frequent distractions, and some topics did not translate well to a virtual environment. Residents from Thailand, which was hit hard by the pandemic and had a significant shift in psychiatric training to online sessions, also reported mixed experiences. Although all residents had good results and passed their examinations, they felt that studying online and the uncertainty with virtual psychotherapy were major inconveniences in their training [94]. In another study, residents were ambivalent. They perceived face-to-face teaching to be superior, but majority of them did not think a complete return to in-person learning would be the most effective option when this becomes possible, implying a preference to continue with some online components in the training [95].

A summary of the key benefits and limitations of the 5 different online-based and technology-assisted educational methods (videoconference, online module/e-learning, virtual patient, software/applications, and social media) is provided in Table 1.

**Table 1:** Summary of the implementation of online-based and technology-assisted psychiatric education.

Method	Key benefits	Limitations
Videoconference	Flexible and applicable for various objectives (lectures, skills training, psychotherapy supervision, etc) Accommodates different instructional strategies (direct, indirect, interactive, and experiential) Convenience of attending sessions regardless of location or schedule	Relies on the internet speed Struggles with nonverbal cues Frequent distractions and Zoom fatigue
Online module	Allows self-paced learning Numerous relevant materials can be designed and included (animations, prerecorded videos, and quizzes) Possibility of reaching a wide range of audiences via a MOOC^a^	Lacks direct supervision More suitable for theoretical aspects of training than clinical aspects Often requires collaboration and resources to design the modules
Virtual patient	Valuable for learning uncommon cases Engaging learning experience	Requires high levels of resources for development Can be frustrating to interact in case of speech recognition issues
Software/applications	Serves as a convenient point of reference Integration with a smartphone Highly favored in checking medication dosing and information	Requires certain levels of resources for development Software that primarily acts as a gateway for institutional logins to certain websites is not often used
Social media	Promotes a continuous learning opportunity Possibility of greater dissemination of knowledge to a larger audience Provides information on current evidence-based studies	Reported knowledge gain that translates into clinical practice is still less significant Relies on the effort of the individual to follow the account and review the shared resource

^a^MOOC: massive open online course.

## Discussion

### Summary of the Results

**Figure 6:**
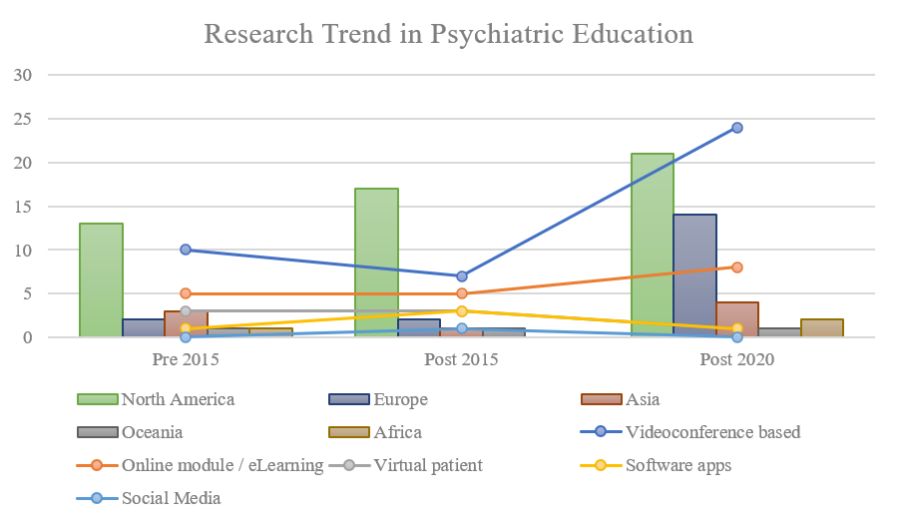
Trend of online-based and technology-assisted psychiatric education in the 3 study phases (prior to 2015, 2015-2019 [prepandemic], and 2020 onward [postpandemic]).

The findings across the 3 phases (prior to 2015, 2015-2019 [prepandemic], and 2020 onward [postpandemic]) illustrated the creative integration of online-based and technology-assisted learning in psychiatric education for trainees (Figure 6). Five approaches were identified: videoconference, online module/e-learning, virtual patient, software/applications, and social media. These methods were used for various objectives, including but not limited to teaching theory knowledge, skills training, psychotherapy supervision, and information retrieval. North American countries were leading in research output, followed by European countries. There was consistent research presence from countries in Asia, Africa, and Oceania throughout the 3 phases, with a slight increase after 2020.

The trend of online-based and technology-assisted psychiatric education showed changes from one phase to another. Videoconference-based learning was consistently the preferred way of integrating technology into the learning or training of trainees. Meanwhile, there was a growing trend of online module/eLearning platform use, virtual patient use, and development of applications or software from the “prior to 2015” phase until the prepandemic phase (2015-2019). The trend however stagnated in the postpandemic phase. Videoconference-based learning was more dominantly used due to 3 factors. First, as seen in other fields, the sudden shift to online was not accompanied by the readiness of other technology modalities to assume the responsibility to continue the learning process [96]. Second, the improved accessibility and availability of videoconference platforms, such as Zoom, Google Meet, and Microsoft Teams, served as a plus point [97], and less resources were needed to shift learning to those platforms rather than developing online modules in an expedited manner. The third factor corresponds to the nature of psychiatric learning, which is preferably face-to-face, but among the choices that were presented, videoconference-based learning was a method that may offer some compromise in terms of allowing direct and synchronous instructional methods despite physical distance [98].

In terms of didactic teaching, evidence showed that online-based or technology-assisted learning is beneficial and well-tolerated by residents. Barring minor issues pertaining to connectivity, a recurring problem reported by several studies [59,65,78], theoretical learning of core knowledge in psychiatry through online platforms has been helpful in improving knowledge, and the impact cannot be understated. Some trainees or residents even expressed their preference for receiving academic or continuous professional development activities virtually, citing convenience and ease of access as important factors that encourage participation despite their busy schedules [72,93]. Nevertheless, psychiatry is not purely a theoretical field in medicine. Psychiatry knowledge must be paired with competency in necessary skills, such as communication skills and psychotherapy skills, to ensure robust and quality training. Learning these skills through an online platform is possible, and a study showed that learning CBT online was not inferior to learning it through in-person training [90]. Nonetheless, proper hands-on guidance remains necessary for mastering these skills, as it might not be possible to fully replicate or demonstrate those subtle, nuanced techniques through a screen. Hence, harmonizing virtual theoretical learning and practical hands-on learning to develop comprehensive blended learning may be a more interesting proposition [82]. Nevertheless, it must be highlighted that the approach of providing knowledge about certain skills and evaluating knowledge levels in trainees is vastly different from teaching skill competencies and then evaluating the levels of competencies of trainees. Because of this issue, some practitioners preferred in-person learning to ensure an optimum level of skill attainment, as compared to through online learning.

As demonstrated by many studies successfully integrating simulation in their medical training, the virtual patient concept (essentially simulating the experience of seeing a patient with the assistance of technology) has been quite helpful in psychiatry education. This concept has a unique strength: the ability to simulate cases that may be uncommon in clinical practice [99]. For example, a study used this concept to portray a refugee with PTSD symptoms, enriching the training of psychiatric trainees and increasing their confidence in managing PTSD cases [48]. Adjacent to the virtual patient concept is the virtual reality (VR) concept. VR allows for an immersive experience, frequently described as “being there,” which involves more senses beyond just sight [100]. It has been used as part of the training curricula in medical fields, including orthopedics [101], surgery [102], and ophthalmology [103], with varying degrees of success. In psychiatric services, VR has been implemented as part of therapy or treatment, for example, exposure therapy for phobic disorders [104] and social skills training in patients with autism spectrum disorder [105]. Unfortunately, VR has not been extensively used in psychiatric education yet, perhaps due to the limitation of the current technology in grasping the complexity of psychiatric cases. As technology rapidly evolves, it remains an exciting avenue to explore in the future.

Competency is often assessed through an examination process. During the pandemic, the transition to online examinations became common worldwide, and psychiatry was no different. Online assessments were applied to various aspects of the psychiatry curriculum. While there were few issues with online theory examinations [86], the same cannot be said for other aspects. For example, an article described the challenges in sitting for the virtual Clinical Assessment of Skills and Competencies (CASC) under the Royal College of Psychiatrists, United Kingdom, where constant worry about internet issues, a sense of disconnect, and an inability to mentally reset between stations affected performance and the overall experience [87]. Another article described the experience of the online Basic Specialist Training examination under the College of Psychiatrists of Ireland [89]. Despite acknowledging the superiority of face-to-face examinations, the online examination was described as nearly as good and more favorable in view of respondents being in a familiar environment as well as saving cost and time to travel [89]. Although it is not possible to compare these examinations directly, the Basic Specialist Training examination highlighted that such examinations could be conducted virtually, but thorough preparation and strong technical support are warranted.

Another interesting aspect of how technology can be valuable in psychiatric training is through the development of cultural competence. Cultural competence refers to the capacity to respond to the unique needs of the population. In the context of psychiatry, it refers to the development of knowledge, skills, and attitude, which can enable the formulation of an intervention that considers the sociocultural backgrounds and sensitivities of psychiatric patients. In turn, this allows for a comprehensive and tailored treatment for patients, especially those from racial and minority ethnic groups. Previous measures to promote cultural competence included learning trips and student exchange programs. However, technology can also offer interesting and possibly cheaper options to achieve the same goals. Trinh et al [80] described an online module program to promote culture sensitivity in a psychiatry department. Three modules were developed, including presentation slides, case vignettes, and recorded videos, covering important topics, such as DSM-5 Outline for Cultural Formulation, Cultural Formulation Interview, and cultural identity as a multidimensional construct. This program was initially met with surprising feedback, with most clinicians indicating that they were not familiar with what questions to ask to elicit a cultural history; however, after completion, the respondents endorsed the module as useful and reported that they would change their practice, suggesting that a brief online module may have potential in this area.

Exploring the application of online-based or technology-assisted learning in psychiatry education for trainees holds significance in the training of future psychiatrists, especially in low- and middle-income countries (LMICs). To put this in context, most African countries have a massive shortage of psychiatrists, with an average of 0.1 per 100,000 people in the 47 countries across the World Health Organization African region [106]. Moreover, both Liberia [107] and Timor-Leste [108] had only 2 trained psychiatrists, indicating the pressing need to support the mental health systems in these countries. While there is no easy fix for this situation, efforts to increase the number of psychiatrists is of utmost importance. In this context, a concerted and collaborative effort among universities or training programs across regions or continents for the training of future psychiatrists in LMICs is needed to alleviate this issue, and the use of online platforms could be the key to bridging the gap.

### Limitations

Our review has some limitations. First, we did not limit the types of publications, resulting in variations in the quality and rigor of the studies. Additionally, majority of the articles were from Western countries, with very few from LMICs, which might reduce generalizability. It is important to note that while a healthy number of studies were included in this scoping review, ultimately obvious heterogeneity was present in terms of the outcomes measured. Majority of the studies had a 1-group intervention study design and had a rather small sample size. Therefore, while some of the included studies might have shown positive responses or outcomes, the findings need to be interpreted carefully in the context of these factors, which might affect generalizability. Moreover, the lack of well-designed comparative intervention studies limits the understanding of the effectiveness of online learning in comparison to traditional face-to-face learning. Another key limitation is the profound lack of an objective assessment as part of the outcome measure within the included studies. Most of the studies assessed satisfaction and attitudes toward the interventions, rather than the actual impact of the interventions.

Moving forward, more well-designed studies in psychiatric education for trainees are needed, especially with objective assessments, to truly evaluate the suitability of online-based and technology-assisted learning. There is a clear paucity of studies evaluating the efficacy of psychotherapy skills training delivered virtually, which may be of significance to LMICs that need a higher number of competent mental health professionals. Lastly, the emergence of AI systems, such as ChatGPT and DeepSeek, can be a game changer for the psychiatric education of trainees, and further exploration is required on how to maximize the benefits of these systems while developing safe and competent psychiatrists in the future.

### Conclusion

Videoconference-based learning was the most widely implemented approach, followed by online modules and virtual patients. Despite the outcome heterogeneity and small sample sizes in the included studies, the application of such approaches may have utility in terms of knowledge and skills attainment. With further fine-tuning, these approaches could become effective solutions to address the significant deficiency of psychiatrists, especially in LMICs.

## Data Availability

The data supporting the findings of this study are available from the corresponding author upon reasonable request.

## Appendix

Multimedia Appendix 1 Search strategies.

Multimedia Appendix 2 Data of the 3 study phases.

Multimedia Appendix 3 PRISMA (Preferred Reporting Items for Systematic Reviews and Meta-Analyses) checklist.

## Abbreviations

AI: artificial intelligence
CBT: cognitive behavioral therapy
DSM: Diagnostic and Statistical Manual of Mental Disorders
LMIC: low- and middle-income country
MOOC: massive open online course
OSCE: objective structured clinical examination
PTSD: posttraumatic stress disorder
RANZCP: Royal Australian and New Zealand College of Psychiatrist
SRQR: Standards for Reporting Qualitative Research
VR: virtual reality
