# Supplementary material for: Online-Based and Technology-Assisted Psychiatric Education for Trainees: Scoping Review
Source: JMIR Med Educ. 2025 Apr 15;11:e64773. doi: 10.2196/64773 (PMC12041828; doi:10.2196/64773)
Supplement: Multimedia Appendix 1 [file mededu_v11i1e64773_app1.docx]

**Multimedia Appendix 1.** Search strategies.

PubMed (658):

(("resident*"[Title/Abstract] OR "trainee*"[Title/Abstract] OR "postgrad*"[Title/Abstract] OR "graduate*"[Title/Abstract]) AND ("psychiatr*"[Title/Abstract] OR "psychologic* medicine"[Title/Abstract]) AND ("education"[Title/Abstract] OR "training"[Title/Abstract] OR "development"[Title/Abstract] OR "learning"[Title/Abstract] OR "teaching"[Title/Abstract] OR "internship"[Title/Abstract] OR "traineeship"[Title/Abstract] OR "residency"[Title/Abstract] OR "course"[Title/Abstract] OR "lesson"[Title/Abstract] OR "program"[Title/Abstract] OR "programme"[Title/Abstract] OR "class"[Title/Abstract] OR "workshop"[Title/Abstract] OR "module"[Title/Abstract] OR "mooc"[Title/Abstract] OR "academic"[Title/Abstract] OR "clerkship"[Title/Abstract] OR "curriculum"[Title/Abstract]) AND ("on-line"[Title/Abstract] OR "online"[Title/Abstract] OR "digital"[Title/Abstract] OR "virtual"[Title/Abstract] OR "internet-based"[Title/Abstract] OR "internet based"[Title/Abstract] OR "web-based"[Title/Abstract] OR "web based"[Title/Abstract] OR "telepsychiatry"[Title/Abstract] OR "tele-psychiatry"[Title/Abstract] OR "cyber"[Title/Abstract] OR "electronic"[Title/Abstract] OR "e-learning"[Title/Abstract] OR "tele-education"[Title/Abstract] OR "videoconferencing"[Title/Abstract] OR "elearning"[Title/Abstract] OR "distance"[Title/Abstract]))

ERIC (86):

(online OR on-line OR digital OR virtual OR internet-based OR internet based OR web-based OR web based OR internet OR videoconferencing OR elearning OR e-learning OR telepsychiatry OR tele-psychiatry OR distance) AND (development OR internship OR residency OR course OR module OR clerkship OR curriculum OR teaching OR training OR education OR learning OR academic) AND (residents OR trainees OR postgraduates OR graduates OR graduate) AND (title:psychiatry OR title:psychiatric)

Cochrane (168):

("online" OR "on-line" OR "digital" OR "virtual" OR "internet-based" OR "internet based" OR "web-based" OR "web based" OR "internet" OR "telepsychiatry" OR "elearning" OR "e-learning" OR "videoconferencing" OR "distance"):ti,ab,kw AND ("psychiatry" OR "psychiatric" OR "psychological medicine"):ti,ab,kw AND ("education" OR "training" OR "learning" OR "development" OR "teaching" OR "internship" OR "traineeship" OR "residency" OR "program" OR "programme" OR "module" OR "clerkship" OR "curriculum" OR "MOOC" OR "academic"):ti,ab,kw AND ("residents" OR "trainees" OR "postgraduates" OR "graduate" OR "graduates"):ti,ab,kw

Scopus (874):

TITLE-ABS-KEY("residents" OR "trainees" OR "postgraduates" OR "graduates") AND TITLE-ABS-KEY("psychiatry" OR "psychiatric" OR "psychological medicine") AND TITLE-ABS-KEY("education" OR "training" OR "development" OR "learning" OR "teaching" OR "internship" OR "traineeship" OR "residency" OR "course" OR "lesson" OR "program" OR "programme" OR "academic" OR "class" OR "workshop" OR "module" OR "mooc" OR "clerkship" OR "curriculum") AND TITLE-ABS-KEY("on-line" OR "digital" OR "virtual" OR "internet-based" OR “internet based” OR "web-based" OR “web based” OR "telepsychiatry" OR “tele-psychiatry” OR "cyber" OR "electronic" OR "e-learning" OR "tele-education" OR "videoconferencing" OR "online" OR "elearning" OR "distance")

PsycInfo (448)

AB ( "online" OR "on-line" OR "digital" OR "virtual" OR "internet-based" OR "internet based" OR "web-based" OR "web based" OR "internet" OR "telepsychiatry" OR "elearning" OR "e-learning" OR "videoconferencing" OR "distance" ) AND AB ( "psychiatry" OR "psychiatric" OR "psychological medicine" ) AND AB ( "education" OR "training" OR "learning" OR "development" OR "teaching" OR "internship" OR "traineeship" OR "residency" OR "program" OR "programme" OR "module" OR "clerkship" OR "curriculum" OR "MOOC" OR "academic" ) AND AB ( "residents" OR "trainees" OR "postgraduates" OR "graduate" OR "graduates" )
