# Supplementary material for: Online-Based and Technology-Assisted Psychiatric Education for Trainees: Scoping Review
Source: JMIR Med Educ. 2025 Apr 15;11:e64773. doi: 10.2196/64773 (PMC12041828; doi:10.2196/64773)
Supplement: Multimedia Appendix 2 [file mededu_v11i1e64773_app2.docx]

**Multimedia Appendix 2.** Data of the 3 study phases.

Table S1. Prior to 2015

| No. | Authors, Year, Country | Aim / Objectives | Methods | Measures | Outcome key findings | Assessment of the study | MERSQI Scoring |
| --- | --- | --- | --- | --- | --- | --- | --- |
|  | Powsner and Byck, 1991, United States [1] | To describe components of The Electric Resident System and actual experiences during system implementation. | Computer software for documentation, drug information search and Medline search. | -The usage log of Grateful Med and Drug Interactions Program  -Anonymous survey | Significant positive correlation between the number of consults and the number of MEDLINE searches.  All residents indicated positive changes in terms of computer experience. | - Educational case report - One-group posttest-only design - Subjective measures. | 6 / 18 |
|  | Gammon et al, 1998, Norway [2] | To assess the effect of video-conferencing on the quality of psychotherapy supervision | Alternated videoconference-based and face-to-face psychotherapy supervision. | -Semi-structured interview | Major concerns with non-verbal cues.  Positive effects in terms of psychotherapy supervision and better preparation as not required to travel far, and having neutral space away from supervisor’s office. | - One-group posttest-only design - Subjective measures. - Small number of participants. - No triangulation, member checking or reflexivity. | (SRQR: 14/21) |
|  | Walter et al, 2004, United States [3] | To assess the utilization of video-conferencing and web-based course management system as educational tool for psychiatry residents. | Videoconference seminars | Questionnaire  - Quality of technology rated on a Likert scale.  - Qualitative feedback | Participants with slower internet had less satisfaction with the lower sound quality.  No significant difference in picture and audiovisuals quality. | - One-group posttest-only design - Subjective measures. - Small number of participants. | 7 / 18 |
|  | Rahman et al, 2006, Pakistan [4] | To test the feasibility of providing training and supervision in child psychiatry. | Store and forward email with case summary | Simple qualitative feedback | CAPSIG members perceived improvement in their diagnostic and management skills. | - Educational case report - Subjective measures. - More to anecdotal feedback. - Possible response bias to respond positively to their own center initiative. | NA |
|  | Briscoe et al, 2006, United States [5] | To gather feedback from medical students and residents in training regarding their perceptions of technology in medicine. | Survey on application of technology in medicine | Web-based survey with 44 questions (Likert style) | 96% participants agreed that “Technology skills are important in medical training”.  51% preferred printed material when initially learning a topic, however, when referencing topic that has been learned, 49% preferred digital media.  Majority of the residents preferred internet-based resources when providing patient care. | - Cross-sectional survey. - Subjective measures. - Not exclusively in psychiatry trainees. | 6.5 / 18 |
|  | Greenwood and Williams, 2008, Australia [6] | Educational meetings for psychiatrists and trainees in rural locations to decrease professional isolation and to obtain continuing professional development (CPD) credit. | Videoconference seminars | -Online quantitative survey.  -Qualitative data from online survey and phone interview. | Participants preferred to attend the seminars from remote sites in view of logistic issue.  83% of respondents found all six seminars beneficial, while 84% agreed that seminars via videoconferencing reduced feelings of professional isolation. | - One-group posttest-only design - Subjective measures. - Included psychiatrists and psychiatric trainees. - No triangulation, member checking or reflexivity. | 7 / 18 |
|  | Kenny et al, 2008, United States [7] | To improve interviewing skills and diagnostic acumen through practice with a female adolescent virtual human with post-traumatic stress disorder. | Virtual patient system for PTSD | -Virtual Patient Pre- and Post-Questionnaire  -Justina Pre- and post-questionnaire | Participants rated the believability of the system to be 4.5/7, and ability to understand the patient, 5.1/7.  Participants rated the system at 5.3 as frustrating to talk to, due to speech recognition problems, or inappropriate responses. | - One-group posttest-only design - Subjective measures. - Included medical students, psychiatric trainees and psychiatric fellows. - Scales developed by authors were not validated before. | 6 / 18 |
|  | Kulier et al, 2008, (Germany, Hungary, Spain, Switzerland, United Kingdom) [8] | The authors developed an e-Evidence Based Medicine course, translated it into various languages and carried out an evaluation across five European countries. | e-Learning modules for evidence based medicine | -Pre-and post-questionnaire to measure knowledge and attitude. | Significant knowledge gain in all five modules. | - One-group pretest-posttest design - Objective measures. - Included trainees from different specialties. - Authors used validated questionnaire. | 14 / 18 |
|  | Szeftel et al, 2008, United States [9] | To assess the impact of a telepsychiatry rotation on the clinical skills and knowledge of psychiatric residents and fellows. | Telepsychiatry | -Questionnaire to assess skills and psychiatric knowledge | Significant increases in self-assessment and improvements in their knowledge level. | - One-group posttest-only design - Objective measures. - Questionnaire developed by authors was not validated. - 92 questions, possible response bias / extreme responding. | 10 / 18 |
|  | Bayar et al, 2009, Turkey [10] | To investigate the efficacy of a web-based mental disorder stigma education program for residents and psychiatrists. | Informative email on stigma education | -Questionnaire with Likert scale on stigmatizing attitude, social distance scale | Participants from experimental group had significantly higher overall questionnaire scores (lesser stigmatizing attitudes) as compared to the control group. | - Randomized controlled trial - Objective measures. - There was no baseline level to compare to evaluate the effectiveness. - The social distance scale has acceptable reliability. - Unclear whether the lesser stigmatizing attitude contributed by the informative email. | 11 / 18 |
|  | Garside et al, 2009, Canada [11] | To assess the efficacy of an e-learning module on the Form 1. | e-Learning module on Form 1 | -Assessment A and B (questions assessing knowledge & clinical reasoning | Statistically significant improvement in terms of knowledge, clinical reasoning and ability to fill out the form 1. | - One-group pretest-posttest design - Objective measures. - Small number of participants. - Not exclusively for psychiatric residents. - Assessments had excellent inter-rater reliability. | 11 / 18 |
|  | Gorrindo et al, 2011, United States [12] | To describe a web-based computer simulation tool assessing physician competence in obtaining informed consent before prescribing antipsychotics to a simulated patient with psychosis. | Web-based simulation software for psycho-pharmacology | -Physician performance – based on the number of required elements completed.  -Confidence Scale  -System Usability Scale | Participants scored a mean of 4.4 / 5 for physician performance.  Statistically significant improvements were found on all four items of the Confidence Scale.  Participants reported a high level of usability, a median score of 86.5 on the System Usability Scale. | - One-group pretest-posttest design - Objective measures. - Small number of participants. - They used a modified Confidence Scale to rephrase the wording – not validated. | 11.5 / 18 |
|  | Pignatiello et al, 2011, Canada [13] | To describe the implementation of The Telelink Mental Health Program | Telepsychiatry | -Program evaluation forms. | 82% of the residents found the telepsychiatry experience as interesting and 78% were interested in participating further in telepsychiatry.  The residents found the experience interesting and enjoyable. | - One-group posttest-only design - Subjective measures. - There was no information regarding the evaluation forms. | 7 / 18 |
|  | Chipps et al, 2012, South Africa [14] | To evaluate the use of videoconferencing in a Psychiatry  Education Program delivered via videoconferencing. | Videoconference-based seminar | -Evaluation questionnaire using Likert scale, assessing implementation process and the participants’ satisfaction with videoconferencing as education tool. | Videoconferencing was perceived as an excellent education tool and an appropriate tool to teach psychiatry by more than half the registrars.  Only 39% indicated that videoconferencing was as effective as face-to-face teaching.  Although registrars reported moderate satisfaction with the videoconference-based education program, there were decrease of interest to attend further videoconference in the future.  There was significant dissatisfaction with the audio quality and, but registrars were satisfied with picture and PowerPoint slides quality. | - One-group posttest-only design - Subjective measures. - One-third of participants (9/27) did not participate in the evaluation (possible attrition). - The evaluation questionnaire was reliable and internally consistent. - No assessment to compare knowledge and skill outcome. | 8.5 / 18 |
|  | Pataki et al, 2012, United States [15] | To investigate the feasibility of interviewing a virtual adolescent patient (VP) with posttraumatic stress disorder (PTSD) | Virtual patient on PTSD | -Tellegan Absorption Scale (Pre)  -Immersive Tendencies Questionnaire (Pre)  -Questionnaire assessing experience with virtual reality (Pre).  -Brief test of knowledge of PTSD based on DSM-IV TR (Pre and post)  -Presence Questionnaire (Post)  -Self-report questionnaire on believability of virtual patient and quality of experience (post) | Trainees’ anticipation of experience with the VP closely matched their actual experience with the pre-interview TAS and ITQ scores positively correlated with PQ scores.  Trainee mean scores were: Believability of System (4.5/7), Understanding the VP (5.1/7), and Frustrating to Interview (5.3/7).  Lack of change pre-and post-test on PTSD knowledge.  Trainees expressed satisfaction in being understood and eliciting responses from Justina despite frustration when Justina at times was unable to understand questions. | - One-group posttest-only design - Objective and subjective measures. - Not specifically in psychiatric trainees. - Small number of participants. - Possible response bias to respond positively to their own center initiative. - The scale developed by the authors to assess change in knowledge may not be valid to detect change. | 10.5 / 18 |
|  | Dzara et al, 2013, United States [16] | To better understand how psychiatry residents and medical students evaluate the training provided in the telepsychiatry rotation. | Telepsychiatry | -Pilot survey using Likert scale, multiple response checkboxes and open-ended comments to evaluate the experience and attitude toward telepsychiatry | All agreed with the statements “Participating in the telepsychiatry clinic was a good use of my clinical training time,” and “Using telepsychiatry enhanced my overall training in psychiatry.  All residents agreed that “The direct supervision I received during my telepsychiatry rotation helped provide a com- prehensive learning experience.”  60% of trainees disliked the technical difficulties and confusion in reading patient’s affect.  Others noted that they disliked the “poor patient / physician relationship /rapport” and that telepsychiatry is more useful in patients who are stable. | - Cross-sectional survey. - Subjective measures. - Small number of patients. - Not a validated scale. - No pre- and post-test assessment. - Possible response bias to respond positively to the supervising psychiatrists | 7 / 18 |
|  | Volpe et al, 2013, Canada [17] | To identify rewarding and challenging aspects from the perspective of psychiatrists and residents participating in tele video consultation services. | Telepsychiatry | -Quantitative: Survey about experience with Telelink  -Qualitative: In depth semi-structured interview and focus group | 68% indicated that telepsychiatry is important and recommend it to be mandatory for training.  72% were at least comfortable in conducting telepsychiatry using Telelink.  76% agreed that the technology was secure, 33.3% concerned with the safety & liability.  From the focus group, they identified 4 main factors:  i. Friendly staff, welcoming environment, comfortable set-up as influencing factors to participate.  ii. Shared values to contribute to rural communities.  iii. Unique training opportunities.  iv. Appreciate the opportunity to expand their practice without having to take on new patients. | - One-group posttest-only design - Objective and subjective measures. - Small number of participants, only 32.5% responded to the survey (26/80). - The focus group only involved 4 psychiatry residents. - Possible response bias to respond positively to their own center initiative. | NA |
|  | Esfahani et al, 2014, Iran [18] | To assess effectiveness of communication skills training program as a distant learning  method in improving empathy. | Videoconference-based empathy skill training and face-to-face empathy skill training | -Jefferson Scale of Empathy  -Objective assessment of empathy.  -Jefferson Scale of Patient’s Perception of Physician Empathy | The score of objective assessment of empathy significantly increased in the attending group.  The score of JSE also increased in the attending group but did not reach a significance level  No increase in empathy was seen in the distance learning group. | - Randomized controlled trial. - Objective measures. - Using validates scales. - Small number of participants (7 for intervention & control respectively) - Stratified random sampling. - The baseline empathy levels between the two groups were unequal. | 13.5 / 18 |
|  | DeBonis et al, 2014, United States [19] | To evaluate the viability of a web-based curriculum in teaching electrocardiogram (EKG) reading skills to psychiatry residents. | e-Learning module on EKG | -Pre- and post-test questions related to the ECG. | Mean improvement was 25 %, with a standard deviation of 18.5 %; mode improvement was 12.5 %.  90% of residents completed it were very interested in receiving additional web-based learning modules in psychiatry education. | - One-group pretest-posttest design - Objective measures. - 22 out 30 (73%) completed the module. - Rather small sample size. - The reason for attrition (8/30) was not clear. | 9.5 / 18 |
|  | DeGaetano et al, 2014, United States [20] | The authors describe their experience developing a 6-month, required training rotation in telepsychiatry. | Telepsychiatry | -Data from VA EMR database.  -Anonymous online survey using Likert scale and open-ended comments to assess strengths and weaknesses of telepsychiatry experience (both trainees and attendings) | Majority of the residents felt comfortable with providing medication management and treating most but not all diagnoses by telepsychiatry.  Half of the residents felt that suicide assessments were difficult through telepsychiatry.  Comments from residents include difficulty to pick-up non-verbal cues, harder to remember patient, new evaluations without a prior visit were difficult.  The attendings agreed that the residents they supervised were comfortable with telepsychiatry.  2 out of 7 attendings agreed that suicide assessments were difficult, concerns about emergency plan, scheduling and equipment problem. | - Educational case report. - One-group posttest-only design - Subjective measures. - Small number of participants. - Non-validated survey. - Possible attrition bias (5/15 did not participate in the evaluation). | 6.5 / 18 |

Table S2. Pre-COVID Pandemic

| No. | Authors, Year, Country | Aim / Objectives | Methods | Measures | Outcome key findings | Assessment of the study | MERSQI Scoring |
| --- | --- | --- | --- | --- | --- | --- | --- |
|  | Pantziaras et al, 2015, Sweden [21] | To assess the impact of training with a virtual patient on confidence in  providing clinical care for traumatized refugee patients. | Virtual patient of traumatized refugee patient. | Harvard Program for Refugee Trauma Confidence Questionnaire | Statistically significant improvements in overall confidence.  Highest improvement was noticed in confidence in identifying and evaluating trauma-related diagnoses and disability, in addition to confidence in treating and managing victims of torture. | - One-group pretest-posttest design - Objective measure. - Rather small sample size, but comparably bigger than the other studies. - Using validated questionnaire. - The study was assessing the confidence, not the clinical competence | 12 / 18 |
|  | Pantziaras et al, 2015, Sweden [22] | To evaluate the impact of training with a virtual patient on the learner’s knowledge of posttraumatic stress disorder (PTSD) | Virtual patient of traumatized refugee patient. | 11 multiple-choice questions regarding  - diagnostic criteria of PTSD according to DSM-IV,  -the management of PTSD  -theoretical aspects of basic communication skills. | The mean post-interaction score was 8.47, which was significantly higher than the pre-interaction score of 7.44. | - One-group pretest-posttest design - Objective measures - Rather small sample size. - No control group to compare the effectiveness of the virtual patient. | 12 / 18 |
|  | Zhang et al, 2015, Canada [23] | To illustrate how the authors designed a delirium education smartphone application and to determine its feasibility as well as the user’s perspectives. | Web-based and smartphone version of the application. | Questionnaire with Likert scale for feedback | Majority of the respondents reported being moderately comfortable with managing delirium prior using the app.  83 % were at least very confident in managing delirium after using the app.  69 % felt the smartphone app as useful in their clinical care for delirious patients. | - One-group posttest-only design - Subjective measures. - Small sample size. - The questionnaire did not assess for competency pre- and post-intervention | 6 / 18 |
|  | Torous et al, 2015, United States [24] | To assess residents’ perceptions and experiences with educational websites. | Cross sectional survey | Survey about psychiatry specific and psychiatry non-specific websites. | Respondents reported that they utilize print resources on average 32 % of the time and electronic resources on average 68 % of the time.  86 % reported they felt there is a need for more psychiatry specific online resources.  79 % noted that online resources should be more visual and interactive.  43 % reported insufficient time, 35 % insufficient faculty guidance, and 43 % cited that resources were not targeted to psychiatry as barriers to use online resources.  UpToDate, PubMed, and Wikipedia were the three most utilized resources by residents. | - Cross sectional survey. - Subjective measures. - Although small number of participants, there was a good participation rate with 92% (57/62) - Only conducted at a single residency program. | 7 / 18 |
|  | Hickey et al, 2015, Canada [25] | To determine if online learning modules can enhance knowledge acquisition and learner satisfaction in psychotherapy education. | A blended course consisting of traditional lectures, online modules and videotape review. | Pre- and post-test for two topics with 20 multiple-choice questions.  Questionnaire with Likert scale to assess learner satisfaction. | Statistically significant knowledge acquisition in each online module and lecture group.  No difference in knowledge acquisition between online modules and lectures.  The residents liked the easy accessibility but they disliked the technical issues. | - Crossover intervention study. - Objective and subjective measures. - Small sample size with 21 residents. - Pre- and post-test questions were similar questions. - The outcome measure was not validated. | 11.5 / 18 |
|  | Teshima et al, 2015, Canada [26] | To explore resident experiences of  telepsychiatry clinical training. | Telepsychiatry | Evaluation form using Likert scale and open-ended comments. | Almost all trainees indicated that the experience was interesting and enjoyable.  97% agreed with the statement “the experience helped me understand more about providing psychiatric services to underserved areas.”  The trainees appreciated the opportunity to learn about different approaches to interviewing clients.  Residents found the technology a “bit unnatural” at the beginning of the session and realized that it was “challenging to interview [patients] at a distance.”  Residents could still successfully “get at non-verbal cues” and “understand some of the technological aspects of doing telepsychiatry.” | - One-group posttest-only design - Subjective measures. - Large sample size with 335 responses and 247 narrative responses. - Continuation of Telelink program earlier publication (Pignatiello 2011; Volpe, 2013) - The qualitative data would be more insightful through interview or focus group. - Possible response bias to respond positively to their own center initiative. | 7 / 18 |
|  | Adeponle et al, 2015, Canada [27] | To conduct an evaluation of impact of a web-based point-of-care information tool and meta-search filter. | Cross sectional survey on various sources of medical Information, as well as analysis of toolkit web page utilization data. | Web-based survey that consisted of  -knowledge of the Psychiatry Toolkit,  -information seeking and perceived search skills,  -attitude towards electronic searching,  -perceived barrier to toolkit use. | 74 % spent up to 1 hour a day for internet biomedical information searches, 19% spent between 1-4 hours.  Top 3 sources that were utilized by respondents: PubMed (58%), UpToDate (35%) and e-Journals (35%).  Common searches included  - medication information (88%),  -treatments for common diseases (65%)  -continuing professional development (42%)  -information on rare diseases (40%)  -diagnosis (37%)  -information for patients (36%).  47% respondents reported using the toolkit. 75% of them reported using the toolkit to answer a clinical question, out of which 86 % reported success finding the answer to their question and the answer influenced their clinical practice. | - Cross sectional survey - Subjective measures. - Small sample size - Single site study - Did not use validated tool - The toolkit appeared to be one stop center to access resource websites via institutional login. - Unclear whether the toolkit or actually the resource websites were the one helping to guide the clinical practice. | 6 / 18 |
|  | Law et al, 2015, Canada [28] | To evaluate results of an online study group (OSG) for geriatric psychiatry continuing professional development. | Asynchronous, expert-facilitated online study group for geriatric psychiatry | Retrospective post- then pre-evaluation using Likert scale in terms of  -self-efficacy, knowledge in geriatric psychiatry  - comfort level with online learning.  Post-test evaluation of effectiveness of instructional methods using Likert scale. | 79 % reported improved efficacy beliefs.  76 % reported improved comfort with online learning.  48 % reported improved perceived knowledge of geriatric psychiatry, 41 % noted there was no change, and 10 % felt that the OSG had a negative impact on their perceived knowledge of psychiatry. | - One-group pretest-posttest design - Subjective measures. - Small sample size, with 29 participants, with only 5 trainees. - The evaluation methods were not validated. - Authors mentioned about thematic analysis of the responses to open-ended questions, but wat not found in the result section. | 7 /18 |
|  | Kupfer et al, 2015, United States [29] | To provide the necessary skill set and support to launch and maintain successful careers in academic psychiatry. | Videoconference-based and face-to-face seminars | Online evaluation of the mentoring sessions | Most participants (14) expressed satisfaction with every bimonthly webinar schedule.  Suggestions to improve the webinar included submissions of questions ahead of the webinar, and to structure the webinars so that 40 minutes for the session, and 20 minutes for networking to get status updates from the class.  Almost all participants felt the program was very helpful. | - One-group posttest-only design - Subjective measures. - Small number of respondents (16). | NA |
|  | Kuhn and Hugo, 2016, United States [30] | The authors presented their residency prolonged exposure web-based training program, PEWeb. | Modules of blended learning on prolonged exposure (bookend blend model). | Mid- and end-of-rotation evaluation sessions. | Residents felt that PEWeb: “the web-training is really effective” and it is “complementary to the seminar.”  Residents appreciated its convenience and availability stating that it is “good that it’s online” and “do-able outside of training.”  One resident stated that “it was a bit overwhelming” in comprehensiveness and detail.  Many residents commented about the videos especially how they helped model the therapy (“trauma interview live demo is so helpful” and “liked that you get to see what it looks like in real time”) and make the training more engaging (“brings it to life” and “moving stories”). | - Educational case report. - One-group posttest-only design - Subjective measures. - Small sample size. - No control to compare to, such as the conventional PE training. | NA |
|  | Wilkening et al, 2016, United States [31] | To evaluate the utility of branched-narrative virtual patients in an interprofessional education series for psychiatry residents. | Branched-narrative virtual patient on advanced psycho-pharmacology. | Online pre- and post-assessments consisted of multiple choice questions and multiple answer queries.  Program evaluation after each session. | The percentage of questions answered correctly significantly improved for sessions 1–3.  Significant improvements in knowledge were not maintained in session 4, but approached significance for content from sessions 1 and 3.  For the first three simulations, the residents agreed that they gained valuable clinical information.  Residents who completed all 4 simulations indicated that they were more confident in prescribing psychotropics and felt their practices improved. | - One-group pretest-posttest design - Objective and subjective measures. - Small number of participants, especially only 4 completed whole program. - No control group to compare to, for example, traditional didactic lectures. | 11.5 / 18 |
|  | Cooper and Roig Llesuy, 2016, United States [32] | To assess and increase physicians’ awareness of catatonia. | Brief e-Learning module on catatonia. | Survey with knowledge questions and Likert scale to describe  -degree of comfort recognizing and managing catatonia,  -the perceived relevance of catatonia to clinical practice. | Significant improvement in correct response rates from 60 to 83% in all participants.  Statistically significant increase in comfort managing catatonia.  All participants rated the 10 minutes module as “just right” in length. | - One-group pretest-posttest design - Objective and subjective measures. - Small sample size due to low completion rates (11 out of 20 psychiatry residents and 9 out of 36 IM residents). - Single site study. - No control group to compare to, for example, traditional lectures or seminars. | 11 / 18 |
|  | Puspitasari et al, 2017, United States [33] | To evaluate the efficacy of the trainer-led online behavioral activation (BA) training program compared to  the self-paced online BA training program. | Trainer-led or self-paced online BA training. | Behavioral activation skills assessment  Reported use of behavioral activation strategies  Training satisfaction questionnaire. | Participants reported high satisfaction with all training sessions regardless of group.    There’s significant increase in behavioral activation skills assessment total scores in both groups.  There were significant group difference favoring trainer-led online group at both post-training and 3 months follow-up.  There’s significant increase in use of behavioral activation strategies in both groups. No significant difference between the two groups. | - Randomized controlled trial - Objective measures. - The use of validated assessment tools. - Unequal training time (360 min in trainer-led group, 160 min in self-paced). - High attrition / dropout rate. - Using intention to treat analysis. | 16.5 / 18 |
|  | Davidson and Evans, 2018, New Zealand [34] | To assess exam preparedness and openness to virtual study groups using a virtual platform to augment OSCE preparation. | Videoconference-based study group for OSCE | Survey with Likert scale and free-text  Verbal feedback on the pilot project after the examination. | 64% at least agreed that they would like a virtual study group to augment exam preparation for OSCEs  42% believed it would decrease isolation and 22% identified networking benefits.  Trainees commented ‘I would feel anxious performing in front of peers, especially if the group was large and consisted of people that I did not know’, ‘Using virtual technology would be too complicated’, and ‘Face-to-face would be essential’.  Participants involved in the pilot project had 100% pass rate. They were unanimous that virtual study group was beneficial as augmentation strategy for OSCE preparation. | - Cross sectional survey and pilot study. - Subjective measure - Small number of respondents (50 trainees for the survey, 4 trainees for the pilot trial) - The feedback on the pilot project might be biased as it was gathered after they have passed their exam. | 6 / 18 |
|  | Reddy et al, 2018, United States [35] | To survey the impact of technological advances on reading preference among psychiatry trainees. | Cross sectional survey on reading behavior or reading style preference. | Survey assessing reading behavior or reading style preference. | Respondents preferred paper (75%) when reading for fun primarily due to the sensory experience (62.5%).  For academic purposes, most participants preferred paper (79.2%) and reported remembering more when they read on paper (91.7%).  Most preferred taking notes on paper (79.2%).  Most respondents preferred the DSM-5 as a book (83.3%).  Residents preferred to have texts with hyperlinks (58.3%) when reading on-screen as hyperlinks helped them understand the article better (62.5%).  Most respondents preferred on-screen reading formats for both checking medication dosing (88.3%) and obtaining detailed medication information (54.2%). | - Letter to the editor - Cross sectional survey - Small sample size (24). - Single site study. | 6 / 18 |
|  | Avery et al, 2018, United States [36] | To examine the impact of an online training module on residents’ attitudes toward people with SUDs. | Online training module on stigma toward individuals with SUDs. | Medical Condition Regard Scale | Attitudes toward individuals with alcohol and opioid use disorders were significantly improved 6 months after the online training module. | - One-group pretest-posttest design - Subjective measure. - Small sample size (35 from IM, 9 from psychiatry, 2 unspecified) - Using validated questionnaire as a proxy to assess the attitude. - There could be confounders which may influence the result within the 6 months period. | 8.5 / 18 |
|  | Dirlam et al, 2018, United States [37] | To describe their experience of Mental Health EMR Tools (MHET) in University of Minnesota Psychiatry Residency | Pre-vetted and curated database | Feedback from the resident. | One of the residents appreciated the clinical support system to provide timely, evidence-based information in a consolidated location that is easily accessible.  Capacity to access information and responses to clinical questions prior to in-person clinical supervision helped to increase confidence. | - Short communication. - Subjective measures. - Anecdotal experience. | NA |
|  | Hameed et al, 2018, Iraq and United Kingdom [38] | To appraise technology-enhanced or web-based learning and the implementation in a conflict zone to support Iraqi trainees in their training (OxPIQ). | Videoconference-based seminars | Feedback from the participants. | The sessions have been associated with improved clinical knowledge and skills of the Iraqi Psychiatry Trainees. | - Narrative of a program with its evaluation. - Subjective measure. - Warrants more objective measures, such as changes in level of knowledge or skills. - Valuable initiative considering it was conducted to promote continuous education in conflict zones. | NA |
|  | Ranjbar et al, 2019, United States [39] | To describe an initiative to develop an integrative medicine curriculum specific to psychiatry | 3 components of integrative psychiatry curriculum: online, experiential, and clinical. | Qualitative feedback during exit interview. | Trainees reported the online curriculum was crucial as part of the IPC to provide quality information linked directly to the published sources. | - One-group posttest-only design - Subjective measure - Small sample size (21). - Possible response bias to respond positively to their own center initiative. | NA |
|  | Williams et al, 2019, United States [40] | To develop and evaluate a 3-hour online webinar‐based course for teaching psychiatry residents about tobacco use disorder. | Three one‐hour webinars in tobacco use disorder. | Pre-test and post-test evaluation on knowledge and survey of attitudes about tobacco use disorders. | Significant increase in knowledge, with a mean score increase of 35.1 points.  There was still a significant increase in knowledge after 3 months compared with baseline, with a mean score increase of 11.4 points, highlighting some knowledge retention. | - One-group posttest-only design - Objective and subjective measures. - Good sample size. - Relatively high completion rate, 152 out of 202 (73.4%) - The evaluation was adapted from validated and reliable source. - Presence of control group can help to compare the effectiveness better. | 11.5 / 18 |

|  | Walsh et al, 2019, United States [41] | To describe the utilization of Twitter as to disseminate educational resources considered helpful in psychiatry training. | 30-min workshop on the professional use of social media, followed by invitation to follow a specified Twitter (@PhippsPsych) account that posted relevant materials for residency training. | Pre-and post-intervention surveys. | Significant increase of participants using Twitter for medical education from 8.2% to 28.6%.  Residents’ ratings regarding usefulness of social media in medical education did not change from pre- to post-survey.  60% reported that the knowledge that they gained from following the account had no impact on their clinical practice, 37.2% reported minimal or average impact, and 2.8% reported great impact.  Ease of access, variety of accounts available to follow, and a convenient way to view up-to-date articles recommended by colleagues were among the positive aspects of Twitter listed by the participants. | - One-group pretest-posttest design - Subjective measure. - Small sample size (49) - Single site. - Difficult to objectively asses the effectiveness - Possible response bias to respond positively to their own centre initiative. | 7.5 |
| --- | --- | --- | --- | --- | --- | --- | --- |

Table S3. Post COVID Pandemic

| No. | Authors, Year, Country | Aim / Objectives | Methods | Measures | Outcome key findings | Assessment of the study | MERSQI Scoring |
| --- | --- | --- | --- | --- | --- | --- | --- |
|  | Samar McCutcheon, 2020, United States [42] | To explore the outcome after implementation of telepsychiatry in outpatient clinic | Telepsychiatry | Survey with Likert scale and free text to assess residents experience with telepsychiatry. | Majority felt that telepsychiatry positively impacted their education experience.  Statistically significant relationship between the perception of telepsychiatry having a positive impact on clinical education.  One resident commented “I did not like performing telepsych visits over the phone. All new patient visits should be via video.” | - One-group posttest-only design - Cross sectional survey - Subjective measure - Small sample size (9 residents) - Single site study. | 7 / 18 |
|  | Rakofsky et al, 2020, United States [43] | A virtual standardized patient-based assessment simulator was developed to  evaluate residents’ proficiency in psycho-pharmacological knowledge and practice. | Virtual patient with symptoms of a treatment-resistant form of major depressive disorder (MDD). | Anonymized residents’ results of PRITE MK5: Somatic Therapies.  Post-test ten-item survey with Likert scale and open text to assess test acceptability | There was a linear trend for greater accuracy within increasing levels of learner experience for all three types of psychopharmacology questions.    The mean total score on the simulator by class correlated significantly with the mean scores on the Somatic Therapies subscale of the PRITE. | - One-group posttest-only design - Objective and subjective measures - Small sample size. - Pilot study has been done prior to the current study. - Since the PRITE results were anonymized, the authors unable to compare the test performance to learners’ real-world outcome. | 9.5 / 18 |
|  | Gargot et al, 2020, France, United Kingdom, Greece, Latvia [44] | To describe implementation of massive open online course (MOOC) for cognitive behavioral therapy (CBT). | MOOC for CBT | Weekly quiz assessing the knowledge on CBT  The rate of completion of the module.  Online survey on the implementation of MOOC. | The average score of 21.4/25 on the first week raised continuously every week up to 23.13/ 25 in the final week.  Despite only 13% completed courses, it was still twice the average retention rate of MOOCs.  Subtitles could be necessary to teach non-native English speakers. | - One-group posttest-only design - Objective and subjective measures. - Large number of participation (7,116), but low completion rate with 13% (954 participants) - Completion of MOOC does not equal to competency in delivering CBT, but may be suitable for delivery of the theoretical knowledge. | 9.5 / 18 |
|  | Wasser et al, 2020, United States [45] | To describe the development, and preliminary impact of two online learning modules designed to teach general psychiatry residents about basic forensic  psychiatry principles. | Online modules on core concepts of confidentiality and duties to third parties. | Pre-test and post-test scores related to forensic concepts. | Statistically significant improvement in knowledge levels in the post test in both modules. | - One-group pretest-posttest design - Objective measure. - Participants were from diverse regions of the United States, but New England residency programs were over-represented. - Small or absent sample sizes from other regions. - Assessing it few months later could help to assess retention of knowledge. | 11.5 / 18 |
|  | Williams et al, 2020, United States [46] | To evaluate treatment practices among psychiatry residents before and 3 months after a course on tobacco use disorder | Three 1-hour webinars on tobacco treatment. | Follow-up survey that assessing attitudes and application of treatment practice, and knowledge about tobacco use disorder | Participants in the follow-up group achieved greater knowledge acquisition.  Residents reported a significant increase in their use of nine of the 12 tobacco treatment practices. | - One-group pretest-posttest design - Low follow-up rates, only 89 responded out of 152 who completed the module. - No control group to compare the effectiveness. | 9 /18 |
|  | Famina et al, 2020, United States [47] | To evaluate the feasibility of early exposure to  tele psychotherapy in residency continuity clinics. | Tele psychotherapy sessions | Unstructured narrative evaluations on the tele-psychotherapy experience. | Minor technical difficulties such as poor connection or brief loss of connection were sometimes experienced but did not affect patient care.  High level of satisfaction reported by the resident physicians.  The attendings felt that the quality of psychiatric care was no different from that provided in regular office settings, and no compromise in the ability of the residents to empathize to the patients.  The residents indicated that the exposure to telepsychiatry benefited their training. | - One-group posttest-only design - Subjective measure - Small sample size, only two residents involved. - The interviews were not synchronously supervised, thus there were questions on the reliability of the assessment on the ability to empathize and the non-verbal cue. | NA |
|  | Beran and Sowa, 2020, United States [48] | To describe the changes and impacts towards clinical training on consultation-liaison psychiatry service during the pandemic | Virtual consultation liaison psychiatry care. | Online survey using Likert scale and narrative feedback on experience to transition to virtual care. | Attendings concerned of reduced quality for the trainees due to change in the supervision experience among the attendings. They also felt trainee supervision and training worse in the pandemic.  Trainees reported moderate satisfaction with virtual consult psychiatry experience but felt less comfortable conducting virtual care and less confidence in their assessments.  Many trainees found video consultations “frustrating”, when attempting to interview patients who had difficulty engaging in virtual interactions (delirium, neurocognitive disorders, mania, etc.). | - Cross sectional survey. - Subjective measures. - Small sample size (4 attendings and 10 residents). - Single site. - Narrative feedbacks were assessed using thematic analysis. | 6.5 / 18 |
|  | Kiing et al, 2021, United States, China, Pakistan, India, Nepal, South Africa,  Zimbabwe, Rwanda [49] | To describe the International Interprofessional Collaborative Office Rounds managing complex developmental and mental health case and its impacts to its participants | Videoconference-based complex case discussion | Baseline enrollment survey to describe the diversity of participants.  General satisfaction survey and impact the program had on their practice using Likert scale at 1 year point. | 41 participants (87%) rated their expectations were “completely” or “mostly” met by the online case discussions.  Mean degree of satisfaction was 3.6 / 4. Only 1 participant rated the sessions as poor.  83% reported the session to have some or definite impact to their practice | - One-group posttest-only design - Cross sectional survey. - Subjective measures. - Multi-countries involvement - Only 47 out of 141 participants (33%) completed the questionnaire – possible response bias. - No objective measures to assess changes in clinical practice. | 7 / 18 |
|  | Cruz et al, 2021, United States [50] | To evaluate the experience and view / concerns of residents, program directors and faculty had with telepsychiatry (TP). | Cross sectional survey | Survey with Likert scales and 47 yes/no or true/false questions about telepsychiatry. | 66% of residents and fellows were interested in TP.  The concerns were the inability to perform a physical exam, unknown liability related to TP, poor Internet connection, TP residency training being insufficient, and certain cultures being less accepting of TP.  Residents and fellows have lesser interest in TP as compared to program directors and faculty. | - Cross sectional survey. - Subjective measures. - Although whole respondent is quite good sample size of 270, residents were only 76, 47 fellows, 57 program directors and 90 faculty members. - The survey questions were adapted from 5 validated questionnaires. | 9 / 18 |
|  | Soll et al, 2021, Germany [51] | To compare the effectiveness of online course in teaching cognitive behavioral therapy (CBT) with in-person training. | Asynchronous, blended, inverted-classroom online learning environment for CBT training (CBT for psychosis) | Satisfaction and Acceptance Questionnaire | Majority of participants in both groups rated the courses as satisfying or very satisfying in all items.  The online training evaluations were non-inferior concerning information content, conception of content, didactic presentation, assessment of the trainer as a suitable role-model, working atmosphere, own commitment, and practical relevance. | - Non-equivalent groups posttest-only design. - Subjective measure - Multi-center site of study - Unequal sample size in online training group (85) and face-to-face group (142). - However, the authors conducted statistical correction to remedy the discrepancy, - No assessment on change of the knowledge level. | 11 / 18 |
|  | Nadeem et al, 2021, Pakistan [52] | To describe and investigate an online trauma curriculum for psychiatry trainees. | Two 3-hours online trauma curriculum. | Summative assessments with multiple choice questions to assess knowledge and clinical skills.  Author-developed scale for skill assessment  Semi-structured qualitative feedback questionnaire | Most of the participants showed competent skills in communication.  All residents scored >50% correct answers in the assessment.  All participants found the online curriculum to be helpful and helped with capacity building while working with trauma survivors.  All participants appreciated the inclusion of video materials and the interactive nature of the sessions.  Few residents thought face-to-face sessions is more helpful | - One-group posttest-only design - Curriculum adaptation due to COVID - Objective and subjective measures. - Small sample size - The scale for the assessment was developed after thorough literature search. | 9.5/ 18 |
|  | Kalayasari and Wainipitapong, 2021, Thailand [53] | A narrative describing the strategies employed in response to the COVID-19 pandemic | Online psychiatric care and training | Results of the national examination.  Survey of the task force. | The national online examination results of all residents were satisfactory.  Trainees concerned with the inconvenience of studying online, decreased number of psychiatric cases for training and new ways of performing psychotherapy. | - Educational case report - Curriculum adaptation due to COVID. - Single-site (Chulalongkorn University) - Objective and subjective measures. - Outcome mentioned in passing. | NA |
|  | Ouanes et al, 2021, Tunisia [54] | To describe implementation of modern technology in psychiatric training in Tunisia | Online psychotherapy workshops with Socrative and Kahoot!  Virtual biostatistics and methodology courses. | Feedback from participants | Participants appreciated the interactive format, and highlighting how live polls encouraged them to participate without the fear of being judged for giving wrong answers.  Participants reported being highly satisfied with the new format of the courses, and many preferred the online to the in-person format.  Only minor occasional drops in connection quality and technical issues with webcams/microphones were reported. | - Educational case report - One-group posttest-only design - Subjective measures - No mention about sample size. - No mention about how the authors received the feedback - Results mentioned in passing. - Innovative idea – gamification of psychiatry knowledge. | NA |
|  | Ranjbar et al, 2021, United States [55] | To investigate the effectiveness of Mind-Body skills to address the burnout issues among psychiatric staffs | Videoconference-based skills training | Survey with Likert scale, yes/no question and open-ended comments | 84% were satisfied with the virtual skills training program.  61% implemented the skills to their patients.  Participants felt the skills taught useful for their own self-care. | - One-group posttest-only design - Subjective measure. - Small sample size (50). - Lacked control group. | 7 / 18 |
|  | Kumar et al, 2021, Malaysia [56] | To evaluate the impact of an alcohol withdrawal training program on knowledge, attitude, and perception among healthcare providers in a hospital setting. | Videoconference-based seminar | Pre- and post-training assessments with a locally validated knowledge, attitude, perception questionnaire on alcohol withdrawal and CIWA-Ar | The post-training scores are significantly higher than the pre-training scores across the knowledge, attitude, perception and CIWA-Ar.  Cohen’s d effect size was 0.75 (large). | - One-group pretest-posttest design - Objective and subjective measures. - The questionnaire was derived from 4 validated assessment tools. - Rather small sample size (36) - Single-site study. - No control group to compare to. | 13 / 18 |
|  | Trinh et al, 2021, United States [57] | To describe the development and impact of an innovative three-part online cultural sensitivity training program. | Online cultural sensitivity modules | Multiple-choice and open-ended survey on cultural competence.  Multiple choice questionnaire on clinician knowledge and comfort using DSM-5 Outline for Cultural Formulation & Cultural Formulation Interview | Participants rated the technology as user friendly.  Participants also endorsed the module as useful and the learning objectives as met.  271 participants (89%) reported that they would change their practice, suggesting that short online modules may have the potential to help improve patient care.  246 participants (77%) could not identify the relevant portions of DSM-5 that offer ways to explore patient’s cultural history. | - One-group posttest-only design - Subjective measures. - Relatively good sample size. - The questionnaires / survey used were not validated. | 6.5 / 18 |
|  | Heldt et al, 2021, United States [58] | To compare perceptions of remote learning versus in-person learning among faculty and trainees at a single institution during the COVID-19 pandemic | Videoconference-based learning | Evaluation survey using Likert scale assessing knowledge, attitude, and skills towards remote learning. | Trainees and faculty perceived in-person learning more positively than remote learning on the majority of items assessed, but remote learning was more convenient compared to in-person training.  Over a third of trainees reported frequent distractions during remote lectures.  Only a minority of trainees and faculty feel that a complete return to in-person learning would be the most effective option when this becomes possible.  Half of the trainees perceived that comprehension and retention of material were either no different or better with remote learning. | - Cross sectional survey - Subjective measure - Relatively low response size. - Single site study | 6 / 18 |
|  | Scheeres et al, 2021, United Kingdom [59] | To describe the experience of MRCPsych theory examination, VSAQ and its role in in online MRCPsych examination | Online theory examination | Feedback from the psychiatry trainees | Trainees concerned about the connectivity issues, possibility of cheating, catering needs to people with learning needs  Trainees generally agreed for digitization of theory examination post COVID, preferably in a test center | - Posttest-only descriptive article - Subjective measure - Transition to online format due to COVID - Results showed the common themes of the feedback. - Simple qualitative analysis | NA |
|  | Chu and Sathanandan, 2021, United Kingdom [60] | To explore the fairness of such an examination, the difference in trainee experience, and the use of telemedicine to consider what might  be lost as well as gained. | Online clinical examination (CASC) | Feedback from the psychiatry trainees | There was concern about the stability of internet connection for the exam.  The sense of camaraderie and shared experience with peers is also lost, with no opportunity to exchange reassurances with fellow candidates as they pass by between stations.  There is a sense of disconnect that exists during and between the clinical stations.  The connection to the patient can feel poor, even when the Wi-Fi is not.  The lack of framework to mentally reset is particularly notable. | - Narrative of the author’s experience of virtual CASC examination - Subjective measure - Valuable insight from the lens of trainees sitting for the exam, but lack generalizability as the experience may be unique to the author. | NA |
|  | Nalan et al, 2022, Canada [61] | To explore how residents described their experience in the virtual Balint group | Videoconference-based Balint group | Focus group with semi-structured interview | Participants appreciated the Balint group, but the virtual nature of the group led to feelings of an abrupt ending to the session even when the session ended on time. | - One-group posttest-only design - Small sample size (3) - Subjective measure - Qualitative content analysis - The interview was recorded. | SRQR: 17/21 |
|  | Elzain et al. 2022, Ireland [62] | To report the experience of online Balint groups in South West Ireland during the pandemic | Videoconference-based Balint group | Electronic survey with quantitative outcomes and open-ended ‘free-text’ responses to rate their online Balint experience | 60% agreed that online Balint help improving their morale, wellbeing, or job satisfaction.  50% agreed online Balint group helped work feel less stressful and provided a cathartic space and helped them to tolerate more difficult feelings.  75% agreed that online Balint group renewed their interest and understanding of patients.  Most of the participants preferred face-to-face Balint, but they preferred online to no Balint.  One participant reported feeling positive about the online group, with no real difference in their engagement between physical and virtual meeting.  Trainees reported that they like the convenience of online as they could login from their cars or offices without worrying about finding a parking space in the hospital. | - One-group posttest-only design - Cross sectional survey - Subjective measures - Small sample size (12) - Comparison with face-to-face Balint group will be more substantive, but COVID limited the possibility. | 7 / 18 |

|  | Chacko et al, 2022, New Zealand [63] | To report the development and feedback on mindfulness-based cognitive therapy (MCBT) informed virtual wellbeing program for trainees. | Videoconference-based skills training | Online questionnaire with quantitative and qualitative data. | Trainees appreciated the sense of connectedness with the facilitator through the videoconference sessions, compared to self-help apps. | - One-group posttest-only design - Subjective measure - Small sample size (14) - Assessment tool was not validated. - Assessment pre-and post-intervention will be helpful to examine the efficacy. | NA |
| --- | --- | --- | --- | --- | --- | --- | --- |
|  | Li et al, 2022, United States [64] | To develop and pilot three simulation scenarios as part of an educational module on health system science (HSS) for resident physicians. | Videoconference-based lecture and simulation training | Pre-assessment survey on experience with HSS curriculum.  Post-intervention survey with Likert scale and qualitative question on experience of the program. | All participants reported perceived learning gains from the three modules.  Participants had generally positive reactions to the session, especially to the interactive role-play format.  However, virtual format can be awkward due to need for turn-taking and limitations in interactivity. | - One-group posttest-only design - Subjective measure - Rather small sample size, 95. Only 24 were psychiatry residents. - The free-text was analyzed according to directed content analysis. | 8 / 18 |
|  | Tapoi et al, 2022, Multi-European countries [65] | Narrative on experiences conducting the first online EPA Summer School on Research 2021. | Videoconference-based seminars | Feedback at the end of the program. | Participants were highly satisfied with the online format and the topic of research methodology. | - One-group posttest-only design - Subjective measure - Sample size 21 – rather small. - Result was mentioned in passing. | NA |
|  | Wortzel et al, 2022, United States [66] | To evaluate the effectiveness of an online learning module regarding the Bush Francis Catatonia Scale (BFCRS) | 1-hour online module on catatonia | Form A – 50 items multiple-choice test on BFCRS, which assessed theoretical knowledge on BCFRS.  Form B – patient scored the standardized patient video using BFCRS, assessing the practical knowledge.  Free response feedback. | Significant improvement in knowledge of catatonia after taking the module.  Modest knowledge attrition with improvements persisted for 3 months.  Many participants preferred more videos of standardized patients with catatonia findings.  Some items of BFCRS remained difficult to differentiate even after the online course. | - One-group pretest-posttest design - Objective and subjective measures - Good sample size (482), but significant attrition rate (54% attrition at posttest, and 56% at 3-month). - The assessment tools were not validated. - Some of the multiple-choice test placed unequal focus on certain BFCRS items. | 12.5 / 18 |
|  | Gratzer et al, 2022, Canada [67] | To discuss the outcomes of Reading of the Week (ROTW), an online education resource for Canadian psychiatrists and psychiatry residents. | Reading of the Week website – summarizing latest psychiatric literature | Survey using Likert scale and open-ended questions. | Respondents reported a very high rate of satisfaction (97%).  ROTW improved participants’ understanding of psychiatry (93%) and informed their practice (83%).  One resident wrote: “ROTW articles are good preparation for my residency training program and future practice as a psychiatrist.” | - One-group posttest-only design - Subjective measure - Good sample size with 332. - Respondents represented various locations across Canada. - Questionnaire was developed according to Moore’s continuing medical education framework. - Qualitative data was analyzed via thematic analysis. | 9 / 18 |
|  | Shanley et al, 2022, United Kingdom [68] | To highlight the impact of the pandemic on acquiring psychotherapy experience in the context of core psychiatry training. | Videoconference-based psychotherapy supervision | Mixed methods of surveys and interviews were conducted | The impact of remote supervision on the attainment of psychotherapy competencies and the receipt of supervision were felt to be limited.  The trainees felt well supported with the virtual supervision, and only minimal disruption from their ward work was noted.  One trainee found the virtual supervision also convenient and flexible in terms of time and logistic.  The supervisor reported occasional internet problems, but it allowed trainees to attend supervision despite on-call duties. | - One-group posttest-only design - Subjective measure - The survey was not validated. - Small sample size, 5 for the survey, 3 for the interview. - The qualitative data was from personal reflections of the authors. | 6 / 18 |
|  | Gentry et al, 2022, United States [69] | To evaluate the feasibility and appropriateness of administering virtual Clinical Skills Evaluations. | Videoconference-based clinical examination | Pre and post questionnaires were given, employing Likert scales and free text comments, on the suitability of virtual CSEs. | Most participants reported that they were able to adequately assess specific CSE items from the ABPN grading form, but only 44% agreed that mental status could be adequately assessed.  All respondents agreed that suicidal and homicidal risks could be adequately assessed through the virtual CSE.  Few residents indicated that virtual CSEs were inadequate for assessing clinical skills.  Residents mentioned the benefit of virtual CSEs include convenience for participants and patients, decreased anxiety, and increased access to patients.  Whereas, there were concerns such as patients having difficulty connecting, difficulty building rapport, difficulty discerning cues, and challenges in assessing gait and motor function. | - One-group pretest-posttest design - Subjective measure - Validated questionnaires - Small sample size (9 residents, 12 faculty examiners), derived from 1 training institution. - No proper qualitative data analysis. - No control group to compare to. | 11.5 / 18 |
|  | Hewson et al, 2022, United Kingdom [70] | The authors designed and delivered simulation training to improve the confidence and competence of junior doctors beginning work in psychiatry. | Face-to-face or videoconference-based seminars, paired with simulation training | Pre and post-questionnaires employing a combination of Likert and free-text response, to assess learner’s confidence in skills relevant to psychiatry. | Statistically significant improvement in the confidence of trainees across all psychiatry skills tested.  Trainees most enjoyed the ‘interactive’ nature of the training and its helpfulness for aiding clinical practice.  Subgroup analyses indicated that the significant improvements in confidence were also seen in the online method group (GP and core psychiatry trainees), except in 2 topics. | - Two-group pretest-posttest design - Subjective measures - The content validity was assessed earlier through pilot testing of the scenarios with few trainees. - Small sample size - Subgroup analyses was done for both groups. - Only 3 psychiatry trainees involved. - The prior experience in psychiatry was not explored. | 11 / 18 |
|  | Blamey et al, 2022, United Kingdom [71] | To investigate whether a psychiatry-specific virtual on-call training program improved confidence of junior trainees in key areas of psychiatry practice. | Videoconference-based lecture and on-call simulation session. | A series of questionnaire at pre- and post-lectures and shifts respectively, assessing their confidence across ten domains, employing Likert scales | Post virtual lecture, participants reported a significant improvement in:  - confidence in performing seclusion reviews  - prescribing psychiatric medications for acute psychiatric presentations  - working with patients in the section 136 suites  - in feeling of preparedness for psychiatric on-call shifts.  Participants reported a significant improvement in nine out of the ten tested domains after completion of the virtual on-call sessions. | - One-group pretest-posttest design - Subjective measure - Small sample size - Questionnaires used were not validated. - Anonymous survey prevented paired analysis of pre-post session questionnaires. - Possible selection bias; fewer trainees attended the virtual on-call sessions suggested they maybe were those least confident. | 11 / 18 |
|  | Knez et al, 2023, Sweden [72] | To explore residents’ and teachers’ perceptions of the digital format of Metis didactic courses for psychiatry residents in Sweden. | Distance-based self-study, classroom-based meeting days for lectures and supervisions, and distance-based examination. | Post-intervention questionnaire given, employing Likert scales, to assess the residents and teachers’ perceptions of digital psychiatry courses. | 46.4 % preferred a return to the original format with classroom-based course meetings.  Respondents < 50 years were more positive in preferring course meetings to continue in a digital format post-pandemic than respondents > 50 years.  Males preferred to return to the original format with classroom-based course meetings compared to females.  Females were more inclined than males to think that the use of interactive tools had a positive effect on learning.  Residents and teachers believed Phase 1 can be improved by video-based lectures, sound-recorded lectures, digital-group discussions or virtual patients.  For phase 2 and 3, two options with highest rates were digital group-discussions and virtual patients. | - One-group posttest-only design - Subjective measure - Survey was developed according to seven-step process of developing questionnaires for educational research by Artino et al, fine-tuned by experts and then piloted. - 53 items for residents, 45 items for teacher, which could explain the low response rate. | 9 / 18 |
|  | Jacoby et al, 2023, United States [73] | To describe the development, implementation, and piloting of an e-learning neuropsychiatry curriculum for psychiatry residents. | Ten-module, interactive e-learning curriculum focusing on the neurocognitive exam and neurocognitive disorders. | Pre and post-surveys of:  - General Practitioner Attitudes  -Confidence Scale for Dementia (GPACS-D)  - a 24-item neuropsychiatry examination  Qualitative feedback was acquired through 4 open-ended items in the survey. | Respondents were less likely to agree with: “managing dementia is more often frustrating than rewarding” and “I feel frustrated because I do not know how to effectively treat people with dementia”.  No shift in overall attitude score and confidence ratings.  Small but significant improvement in the total number of knowledge items answered correctly (14 out of 22).  The interactivity and the asynchronous format were described as among the strength of the module  Technical difficulties and the need for more questions embedded were among the weakness. | - One-group pretest-posttest design - Objective and subjective measures - Validated assessment tools. - Relatively small sample size (n=80), involving two different centers in Brooklyn, New York. - GPACS-D was validated in primary care physicians populations, not psychiatrists - Residents in both centers had unequal protected didactic time to complete the module. | 14.5 / 18 |
|  | Usman et al, 2023, Ireland [74] | To explore the views of psychiatric trainees and examiners on assessment of communication skills during online high stakes postgraduate examinations. | Videoconference-based communication skills examination | Qualitative data from interviews | Candidates found that virtual communication was nearly as good as face-to-face, but latter would enable to build better therapeutic rapport by picking more cues and reciprocate with empathy.  All participants described the experience of the online examination was “better than expected”. Being comfortable in their familiar surroundings helped the candidates to alleviate the exam anxiety.  All candidates favored online version of exam for practical reasons as it avoided the time and cost of traveling and overnight stay, while all examiners preferred to do face-to-face exam.  The candidates expressed that face-to-face exam allowed more fluidity in communication as they were able to pick more nonverbal cues. Online exam needed a lot of preparation as “if you are not experienced in doing video consultations, then you often interrupt each other”. | - Qualitative study. - Subjective measures - Small sample size (14) - Data was analysed according to Braun and Clarke thematic analysis. - Extra attention given to ensure rigor following the criteria suggested by Lincoln and Guba - Triangulation, member checking and reflexivity were conducted. - The interview was held after the candidates received their exam results, possibly influencing their opinion on the online format. | SRQR: 21/21 |
|  | Collin et al, 2023, United Kingdom [75] | To establish doctors’ experience of training in delivering psychodynamic psychotherapy during the pandemic. | An online survey on impact on psychodynamic training during pandemic and then semi-structured interview was conducted. | Online survey and semi-structured interview to evaluate their experience of psychodynamic psychotherapy training during COVID-19 pandemic. | 26.7 % experienced remote supervision, 6.7 % received face-to-face supervision, and 66.7 % had combination of both methods of supervision.  For future supervision, 46.7 % prefer face-to-face, 26.7 % prefer remote supervision, another 26.7 % were unsure.  Many felt that remote supervision was more flexible and allowed more time for other work demands, but some highlighted ‘Zoom fatigue’ and the subtleties of communication being lost during remote supervision.  Some sharing difficulties to identify a private space for the remote supervision. Majority reported it as less engaging compared with face-to-face.  One therapist reported that quality and effectiveness of remote and face-to-face supervision were the same. | - One-group posttest-only design - Subjective measures - Unvalidated questionnaire - Small sample size, 15 for the survey. - The authors concluded that they reached data saturation after 6 interviews. - The interviews were audio recorded and supplemented by notes made during interviews to help identify key themes. - Interviews analyzed using Braun and Clarke six-stage thematic analysis framework. - Methodological triangulation | 9.5  SRQR: 16/21 |
|  | Westcott et al, 2023, Canada [76] | To evaluate the Psychiatry Education through Play and Talk (PEP Talks), a novel virtual medical improv program to improve communication, teamwork, and conflict resolution skills. | Videoconference-based improv sessions. | Mixed methods surveys, recorded debriefings, and a virtual focus group | Participants agreed that the use of Zoom as “invigorating” or “rejuvenating,” and suggested that “it changed the relationship I had with these online interactions…it is not really a virtual care problem…it is the way that we have structured things [in virtual academic settings]”.  All participants remarked that improv connected to their psychiatry training. | - One-group posttest-only design - Subjective measure - Survey was designed based on prior literature. - Small sample size (n=10) - Low participation rates (22%) further reduce the representativeness and validity of the outcomes - Data triangulation was conducted. | 8  SRQR: 17/21 |
|  | Shekunov et al, 2023, United States [77] | A remote psychotherapy curriculum to adapt traditional psychotherapy skills to telepsychiatry settings in COVID pandemic setting. | Videoconference-based lecture | Pre-and post-intervention surveys to assess remote psychotherapy skills and areas for growth | Access (47%) (reduced no-show rates) and the therapeutic process (24%) (benefits in seeing patients’ home environment) were aspects identified as most beneficial in participants’ remote work.  Technology (24%) and patient engagement (29%) often distracted were identified as the greatest challenge. | - One-group pretest-posttest design - Subjective measures - Small sample size (n=28) - Unvalidated questionnaires - No control group to compare to. - Single site study. | 7.5 / 18 |
|  | Owais et al, 2023, Canada [78] | To evaluate the online component of a blended curriculum for psychiatry residents on the use of electroconvulsive therapy (ECT) | A blended learning curriculum with consisted of didactic seminars, two online clinical cases, and hands-on clinical management of late-life depression with ECT. | Post-course knowledge test and Likert scales | 66 % of residents completed the post-course knowledge test with an average score of 80 %.  96 % found the content clinically relevant and the e-learning experience was important / valuable. | - One-group posttest-only design - Small sample size (n=23) - The questions were self-developed. - No baseline pre-test to compare the efficacy of the program. | 8.5 / 18 |
|  | de Cates et al, 2024, United Kingdom [79] | To provide junior psychiatrists with a large-scale platform to critically appraise recent articles published in the BJPsych. | Videoconference-based journal club webinars | Post-journal club survey with quantitative and qualitative assessment | 82 % rated the webinars sessions as ‘very good’ for organization and structure.  98 % were interested in attending another BJPsych Journal Club (98%).  Top 3 improvements suggested was the more time for questions and answers, to have more stable internet, and to speak not too quickly. | - One-group posttest-only design - Subjective measures - Small sample size (n=45) - The respondents were from 3 countries; England, Ireland, & Scotland. | 7 / 18 |
|  | Ben Ammer et al, 2024, United States [80] | To survey residents about their perception of the pandemic's impact on their clinical skills, didactics experience, training preferences, and future career perceptions. | Cross sectional survey regarding general questions, clinical skills, didactic learning, preferences for future education, and psychiatric careers. | Surveys employing Likert scale and open-ended questions on topics such as general questions, clinical skills, didactic learning, preferences for future education. | 18.1% of respondents were less likely to report feeling engaged with the instructor.    Respondents reported several positives of virtual didactics, such as easier to attend, Zoom was engaging, and they enjoyed guest speakers from other institutions; and there was a positive impact on resident wellness.  Other residents reported negative experiences with virtual didactics, such as technology issues, “Zoom fatigue,” and topics that did not translate well to a virtual environment, distracted by clinical responsibilities during didactics and not be able to connect with their peers.  70.7% of respondents preferred a combination of virtual and in-person didactics for future training.  The least preferred option was completely virtual clinical rounds and completely virtual didactics. | - Cross-sectional survey - Subjective measure - Survey was author-developed, not validated. - Fairly small sample size (n=116) - Low response rate with only 6.4% of the population completed the survey and no representation from an almost 22 states, exposed the study to possibility of being unrepresentative of the entire population | 7 / 18 |
|  | Brown et al, 2024, United States [81] | To analyze mental health clinicians’ perspectives on the acceptability of a web-based antiracism intervention. | Online module on antiracism intervention | Semi-structured interview which was then analyzed using inductive coding and thematic analysis. | 67 % enjoyed the digitally delivered demo module, while 58 % suggested web-based content would be better with in-person or online group components.  33 % expressed preference for an online self-directed structure. | - Qualitative one-group posttest-only design - Subjective measures - Small sample size (n=12) - Interviews were recorded and transcribed. - No triangulation, member checking or reflexivity. - Ethnically-inclusive pool of participants - Single site study. | SRQR: 14/21 |
|  | Noori et al, 2024, United States [82] | To pilot a curriculum on digital psychiatry for a residency program and examine the change in learner confidence. | Videoconference-based seminars and role-play sessions | Pre- and post-session surveys to assess their confidence after the didactic sessions using Likert scales and open-ended questions | Statistically significant improvement in terms of residents’ comfort in:  -assessing digital mental health app and patient’s digital health literacy  -in formally recommending a digital health tool to patients  - prescribing a digital therapeutic to their patients.  Residents’ average comfort level did not exceed “somewhat comfortable” in any objective – indicating need for refinement.  Role-playing exercises were helpful in learning to prescribe and recommend apps. | - One-group pretest-posttest design - Subjective measure - Small sample size (n=12) - Single study site. | 9.5 / 18 |

References

1. Powsner SM, Byck R. Implementing a computer system for psychiatric training: The Electric Resident. *Acad Psychiatry*. 1991;15(2):100-105. doi:10.1007/BF03341304

2. Gammon D, Sørlie T, Bergvik S, Høifødt TS. Psychotherapy supervision conducted via videoconferencing: A qualitative study of users’ experiences. *Nord J Psychiatry*. 1998;52(5):411-421. doi:10.1080/08039489850139445

3. Walter DA, Rosenquist PB, Bawtinhimer G. Distance Learning Technologies in the Training of Psychiatry Residents: A Critical Assessment. *Acad Psychiatry*. 2004;28(1):60-65. doi:10.1176/appi.ap.28.1.60

4. Rahman A, Nizami A, Minhas A, Niazi R, Slatch M, Minhas F. E-Mental health in Pakistan: A pilot study of training and supervision in child psychiatry using the internet. *Psychiatr Bull*. 2006;30(4):149-152. doi:10.1192/pb.30.4.149

5. Briscoe GW, Arcand LGF, Lin T, Johnson J, Rai A, Kollins K. Students’ and residents’ perceptions regarding technology in medical training. *Acad Psychiatry*. 2006;30(6):470-479. doi:10.1176/appi.ap.30.6.470

6. Greenwood J, Williams R. Continuing professional development for Australian rural psychiatrists by videoconference. *Australas Psychiatry*. 2008;16(4):273-276. doi:10.1080/10398560801982994

7. Kenny P, Parsons TD, Pataki CS, et al. Virtual justice: A PTSD virtual patient for clinical classroom training. *Annu Rev CyberTherapy Telemed*. 2008;6(1):113-118. https://sites.psychology.unt.edu/~tparsons/PDF/Parsons(Kenny)_Virtual_Justina_PTSD_VP_for_clinical_training.pdf

8. Kulier R, Hadley J, Weinbrenner S, et al. Harmonising evidence-based medicine teaching: a study of the outcomes of e-learning in five European countries. *BMC Med Educ*. 2008;8:27. doi:10.1186/1472-6920-8-27

9. Szeftel R, Hakak R, Meyer S, et al. Training Psychiatric Residents and Fellows in a Telepsychiatry Clinic: A Supervision Model. *Acad Psychiatry*. 2008;32(5):393-399. doi:10.1176/appi.ap.32.5.393

10. Bayar MR, Poyraz BC, Aksoy-Poyraz C, Ankan MK. Reducing mental illness stigma in mental health professionals using a web-based approach. *Isr J Psychiatry Relat Sci*. 2009;46(3):226-230. https://doctorsonly.co.il/wp-content/uploads/2011/12/2009_3_12.pdf

11. Garside S, Levinson A, Kuziora S, Bay M, Norman G. Efficacy of teaching clinical clerks and residents how to fill out the form 1 of the mental health act using an e-learning module. *Electron J e-Learning*. 2009;7(3):239-246. https://academic-publishing.org/index.php/ejel/article/view/1573

12. Gorrindo T, Baer L, Sanders KM, et al. Web-Based Simulation in Psychiatry Residency Training: A Pilot Study. *Acad Psychiatry*. 2011;35(4):232-237. doi:10.1176/appi.ap.35.4.232

13. Pignatiello A, Teshima J, Boydell KM, Minden D, Volpe T, Braunberger PG. Child and youth telepsychiatry in rural and remote primary care. *Child Adolesc Psychiatr Clin N Am*. 2011;20(1):13-28. doi:10.1016/j.chc.2010.08.008

14. Chipps J, Ramlall S, Mars M. Videoconference-based education for psychiatry registrars at the University of Kwazulu-Natal, South Africa. *Afr J Psychiatry*. 2012;15(4):248-254. doi:10.4314/ajpsy.v15i4.32

15. Pataki C, Pato MT, Sugar J, et al. Virtual patients as novel teaching tools in psychiatry. *Acad Psychiatry*. 2012;36(5):398-400. doi:10.1176/appi.ap.10080118

16. Dzara K, Sarver J, Bennett JI, Basnet P. Resident and medical student viewpoints on their participation in a telepsychiatry rotation. *Acad Psychiatry*. 2013;37(3):214-216. doi:10.1176/appi.ap.12050101

17. Volpe T, Boydell KM, Pignatiello A. Attracting Child Psychiatrists to a Televideo Consultation Service: The TeleLink Experience. *Int J Telemed Appl*. 2013;2013:146858. doi:10.1155/2013/146858

18. Esfahani MN, Behzadipour M, Nadoushan AJ, Shariat S V. A pilot randomized controlled trial on the effectiveness of inclusion of a distant learning component into empathy training. *Med J Islam Repub Iran*. 2014;28(1):1-6. https://pmc.ncbi.nlm.nih.gov/articles/PMC4219893/pdf/MJIRI-28-65.pdf

19. DeBonis K, Blair TR, Payne ST, Wigan K, Kim S. Viability of a web-based module for teaching electrocardiogram reading skills to psychiatry residents: Learning outcomes and trainee interest. *Acad Psychiatry*. 2015;39(6):645-648. doi:10.1007/s40596-014-0249-x

20. DeGaetano N, Greene CJ, Dearaujo N, Lindley SE. A pilot program in telepsychiatry for residents: Initial outcomes and program development. *Acad Psychiatry*. 2015;39(1):114-118. doi:10.1007/s40596-014-0122-y

21. Pantziaras I, Fors U, Ekblad S. Innovative training with virtual patients in transcultural psychiatry: the impact on resident psychiatrists’ confidence. *PLoS One*. 2015;10(3):e0119754. doi:10.1371/journal.pone.0119754

22. Pantziaras I, Fors U, Ekblad S. Training with virtual patients in transcultural psychiatry: do the learners actually learn? *J Med Internet Res*. 2015;17(2):e46. doi:10.2196/jmir.3497

23. Zhang MW, Ho RC, Sockalingam S. Methodology of development of a Delirium clinical application and initial feasibility results. *Technol Health Care*. 2015;23(4):411-417. doi:10.3233/THC-150904

24. Torous J, Franzan J, O’Connor R, et al. Psychiatry residents’ use of educational websites: A pilot survey study. *Acad Psychiatry*. 2015;39(6):630-633. doi:10.1007/s40596-015-0335-8

25. Hickey C, McAleer SJ, Khalili D. E-learning and traditional approaches in psychotherapy education: Comparison. *Arch Psychiatry Psychother*. 2015;17(4):48-52. doi:10.12740/APP/60644

26. Teshima J, Hodgins M, Boydell KM, Pignatiello A. Resident evaluation of a required telepsychiatry clinical experience. *Acad Psychiatry*. 2015;40(2):348-352. doi:10.1007/s40596-015-0373-2

27. Adeponle A, Skakum K, Cooke C, Fleisher W. The University of Manitoba Psychiatry Toolkit: Development and Evaluation. *Acad Psychiatry*. 2015;40(4):608-611. doi:10.1007/s40596-015-0419-5

28. Law M, Rapoport MJ, Seitz D, Davidson M, Madan R, Wiens A. Evaluation of a national online educational program in geriatric psychiatry. *Acad Psychiatry*. 2015;40(6):923-927. doi:10.1007/s40596-015-0377-y

29. Kupfer DJ, Schatzberg AF, Dunn LO, Schneider AK, Moore TL, DeRosier M. Career development institute with enhanced mentoring: A revisit. *Acad Psychiatry*. 2015;40(3):424-428. doi:10.1007/s40596-015-0362-5

30. Kuhn E, Hugo E. Technology-based blended learning to facilitate psychiatry resident training in Prolonged exposure (PE) therapy for PTSD. *Acad Psychiatry*. 2016;41(1):121-124. doi:10.1007/s40596-016-0648-2

31. Wilkening GL, Gannon JM, Ross C, et al. Evaluation of branched-narrative virtual patients for interprofessional education of psychiatry residents. *Acad Psychiatry*. 2016;41(1):71-75. doi:10.1007/s40596-016-0531-1

32. Cooper JJ, Roig Llesuy J. Catatonia education: Needs assessment and brief online intervention. *Acad Psychiatry*. 2016;41(3):360-363. doi:10.1007/s40596-016-0632-x

33. Puspitasari AJ, Kanter JW, Busch AM, et al. A randomized controlled trial of an online, modular, active learning training program for behavioral activation for depression. *J Consult Clin Psychol*. 2017;85(8):814-825. doi:10.1037/ccp0000223

34. Davidson D, Evans L. Virtual study groups and online Observed Structured Clinical Examinations practices – enabling trainees to enable themselves. *Australas Psychiatry*. 2018;26(4):429-431. doi:10.1177/1039856218765886

35. Reddy A, Duenas H, Luker A, Thootkur M, Kim JW. Preference of electronic versus paper reading resources among trainees in psychiatry. *Acad Psychiatry*. 2018;42(5):743-745. doi:10.1007/s40596-018-0930-6

36. Avery J, Knoepflmacher D, Mauer E, et al. Improvement in Residents’ Attitudes Toward Individuals with Substance Use Disorders Following an Online Training Module on Stigma. *HSS J*. 2018;15(1):31-36. doi:10.1007/s11420-018-9643-3

37. Dirlam C, Vallera V, Nelson K, Bass D. A Psychiatry Resident’s Perspective of the “Virtual Preceptor” as an Electronic Medical Record Clinical Education Support Tool. *Acad Psychiatry*. 2018;42(5):741-742. doi:10.1007/s40596-017-0880-4

38. Hameed Y, Al Taiar H, O’leary D, Kaynge L. Can online distance learning improve access to learning in conflict zones? The oxford psychiatry in Iraq (OxPIQ) experience. *Br J Med Pract*. 2018;11(2):a1114. https://www.bjmp.org/content/can-online-distance-learning-improve-access-learning-conflict-zones-oxford-psychiatry-iraq-oxpiq-experience

39. Ranjbar N, Ricker M, Villagomez A. The Integrative Psychiatry Curriculum: Development of an Innovative Model. *Glob Adv Heal Med*. 2019;8:2164956119847118. doi:10.1177/2164956119847118

40. Williams JM, Poulsen R, Chaguturu V, Tobia A, Palmeri B. Evaluation of an online residency training in tobacco use disorder. *Am J Addict*. 2019;28(4):277-284. doi:10.1111/ajad.12885

41. Walsh AL, Peters ME, Saralkar RL, Chisolm MS. Psychiatry Residents Integrating Social Media (PRISM): Using Twitter in graduate medical education. *Acad Psychiatry*. 2019;43(3):319-323. doi:10.1007/s40596-018-1017-0

42. McCutcheon S. Putting the cart before the horse: Outcomes following rapid implementation of telepsychiatry in an outpatient resident clinic. *Acad Psychiatry*. 2020;44(6):655-658. doi:10.1007/s40596-020-01316-8

43. Rakofsky JJ, Talbot TB, Dunlop BW. A virtual standardized patient–based assessment tool to evaluate psychiatric residents’ psychopharmacology proficiency. *Acad Psychiatry*. 2020;44(6):693-700. doi:10.1007/s40596-020-01286-x

44. Gargot T, Arnaoutoglou NA, Costa T, et al. Can we really teach cognitive behavioral therapy with a massive open online course? *Eur Psychiatry*. 2020;63. doi:10.1192/j.eurpsy.2020.29

45. Wasser TD, Hu J, Danzig A, Yarnell-MacGrory S, Guzman JR, Michaelsen K. Teaching forensic concepts to residents using interactive online modules. *J Am Acad Psychiatry Law*. 2020;48(1):77-83. doi:10.29158/JAAPL.003890-20

46. Williams JM, Steinberg ML, Wang H, et al. Practice change after training psychiatry residents in tobacco use disorder. *Psychiatr Serv*. 2020;71(2):209-212. doi:10.1176/appi.ps.201900272

47. Famina S, Farooqui AA, Caudill RL. Early use of telepsychotherapy in resident continuity clinics—our experience and a review of literature. *mHealth*. 2020;6. doi:10.21037/mhealth.2019.09.11

48. Beran C, Sowa NA. Adaptation of an academic inpatient consultation-liaison psychiatry service during the SARS-CoV-2 pandemic: Effects on clinical practice and trainee supervision. *J Acad Consult Psychiatry*. 2020;62(2):186-192. doi:10.1016/j.psym.2020.11.002

49. Kiing JSH, Feldman HM, Ladish C, et al. International Interprofessional Collaborative Office Rounds (iiCOR): Addressing Children’s Developmental, Behavioral, and Emotional Health Using Distance Technology. *Front Public Heal*. 2021;9(May 2021):657780. doi:10.3389/fpubh.2021.657780

50. Cruz C, Orchard K, Shoemaker EZ, Hilty DM. A survey of residents/fellows, program directors, and faculty about telepsychiatry: Clinical experience, interest, and views/concerns. *J Technol Behav Sci*. 2021;6(2):327-337. doi:10.1007/s41347-020-00164-5

51. Soll D, Fuchs R, Mehl S. Teaching Cognitive Behavior Therapy to Postgraduate Health Care Professionals in Times of COVID 19 - An Asynchronous Blended Learning Environment Proved to Be Non-inferior to In-Person Training. *Front Psychol*. 2021;12:657234. doi:10.3389/fpsyg.2021.657234

52. Nadeem T, Asad N, Hamid SN, Gul B, Aftab R. Experiences from implementing an lessons from teaching psychiatry trainees at a tertiary care hospital in Karachi, Pakistan. *Asian J Psychiatr*. 2021;65. doi:10.1016/j.ajp.2021.102865

53. Kalayasiri R, Wainipitapong S. Training of psychiatry and mental health in a low- and middle-income country: Experience from Thailand before and after COVID-19 outbreak. *Asia Pac Psychiatry*. 2021;13(4):e12493. doi:10.1111/appy.12493

54. Ouanes S, Larnaout A, Jouini L. Use of modern technology in psychiatry training in a middle-income country. *Asia Pac Psychiatry*. 2021;13(4):e12496. doi:10.1111/appy.12496

55. Ranjbar N, Erb M, Tomkins J, Taneja K, Villagomez A. Implementing a mind-body skills group in psychiatric residency training. *Acad Psychiatry*. 2021;46(4):460-465. doi:10.1007/s40596-021-01507-x

56. Kumar PR, Yee A, Francis B. Impact of alcohol withdrawal training program on knowledge, attitude, and perception among healthcare providers in a hospital setting. *J Subst Use*. 2022;27(1):80-85. doi:10.1080/14659891.2021.1897696

57. Trinh NH, O’Hair C, Agrawal S, et al. Lessons Learned: Developing an Online Training Program for Cultural Sensitivity in an Academic Psychiatry Department. *Psychiatr Serv*. 2021;72(10):1233-1236. doi:10.1176/APPI.PS.202000015

58. Heldt JP, Agrawal A, Loeb R, Richards MC, Castillo EG, DeBonis K. We’re Not Sure We Like It but We Still Want More: Trainee and Faculty Perceptions of Remote Learning During the COVID-19 Pandemic. *Acad Psychiatry*. 2021;45(5). doi:10.1007/s40596-021-01403-4

59. Scheeres K, Agrawal N, Ewen S, Hall I. Transforming MRCPsych theory examinations: digitisation and very short answer questions (VSAQs). *BJPsych Bull*. 2021;46(1):52-56. doi:10.1192/bjb.2021.23

60. Chu K, Sathanandan S. The virtual Clinical Assessment of Skills and Competence: the impact and challenges of a digitised final examination. *BJPsych Bull*. 2021;47(2):110-115. doi:10.1192/bjb.2021.112

61. Nalan P, Manning A. The juice is worth the squeeze: Psychiatry residents’ experience of Balint Group. *Int J Psychiatry Med*. 2022;57(6):508-520. doi:10.1177/00912174221127084

62. Elzain M, Murthy S, Omer S, McCarthy G. Reflective practice in psychiatric training: Balint groups during covid-19. *Ir J Psychol Med*. Published online December 15, 2022. doi:10.1017/ipm.2022.51

63. Chacko E, Vara A, Cheung G, Naskar C, Ramalho R, Bell R. A mindfulness-based cognitive therapy informed virtual psychiatry trainee wellbeing programme: Development and preliminary feedback. *Australas Psychiatry*. 2022;30(5):663-667. doi:10.1177/10398562221119090

64. Li L, Ray JM, Bathgate M, et al. Implementation of simulation-based health systems science modules for resident physicians. *BMC Med Educ*. 2022;22(1). doi:10.1186/s12909-022-03627-w

65. Tapoi C, de Filippis R, Di Lodovico L, et al. 10th EPA Summer School on Research 2021: sharing experience of the first online edition. *Inf Psychiatr*. 2022;98(6):469-474. doi:10.1684/ipe.2022.2443

66. Wortzel JR, Maeng DD, Francis A, Oldham MA. Evaluating the effectiveness of an educational module for the bush-francis catatonia rating scale. *Acad Psychiatry*. Published online January 7, 2022. doi:10.1007/s40596-021-01582-0

67. Gratzer D, Islam F, Sockalingam S, Beckett R. Reading of the Week: a continuing professional development program for psychiatrists and residents that Osler would have liked. *Can Med Educ J*. 2022;13(1):81-85. doi:10.36834/cmej.72089

68. Shanley I, Jones C, Reddi N. Medical psychotherapy training and the COVID‐19 pandemic. *Br J Psychother*. 2022;38(2):338-352. doi:10.1111/bjp.12719

69. Gentry MT, Murray AP, Altchuler SI, McKean AJ, Joyce JB, Hilty DM. Development and implementation of a virtual clinical skills examination in general psychiatry. *Acad Psychiatry*. 2022;47:48-52. doi:10.1007/s40596-022-01691-4

70. Hewson T, Foster H, Sanderson R. Using socially distanced and online simulation training to improve the confidence of junior doctors in psychiatry. *BJPsych Bull*. 2022;47(4):235-241. doi:10.1192/bjb.2022.18

71. Blamey H, Harrison CH, Roddick A, Malhotra T, Saunders KEA. Simulated virtual on-call training programme for improving non-specialised junior doctors’ confidence in out-of-hours psychiatry: Quantitative assessment. *BJPsych Bull*. 2023;47(5):287-295. doi:10.1192/bjb.2022.40

72. Knez R, El Alaoui S, Ivarson J, et al. Medical residents’ and teachers’ perceptions of the digital format of nation-wide didactic courses for psychiatry residents in Sweden: a survey-based observational study. *BMC Med Educ*. 2023;23(1):9. doi:10.1186/s12909-022-03989-1

73. Jacoby N, Gullick M, Sullivan N, Shalev D. Development and evaluation of an innovative neurology e-learning didactic curriculum for psychiatry residents. *Acad Psychiatry*. 2023;47(3):237-244. doi:10.1007/s40596-023-01769-7

74. Usman M, Adamis D, McCarthy G. Perspectives of psychiatric trainees and examiners on the assessment of communication skills during an online clinical examination: A qualitative study. *Ir J Psychol Med*. Published online June 15, 2023. doi:10.1017/ipm.2023.19

75. Collin G, Turner O, Luthra V. Evaluation of doctors’ experience of psychodynamic psychotherapy training at Leeds and York Partnership NHS Foundation Trust during the COVID‐19 pandemic. *Br J Psychother*. 2023;39(3):425-447. doi:10.1111/bjp.12833

76. Westcott S, Simms K, van Kampen K, Jafine H, Chan TM. Off-script, online: Virtual medical improv pilot program for enhancing well-being and clinical skills among psychiatry residents. *Acad Psychiatry*. 2023;47(4):374-379. doi:10.1007/s40596-023-01778-6

77. Shekunov J, Swintak C, Somers K, et al. The virtual couch: A curriculum on the question of the fundamentals of remote psychotherapy—Pilot study. *Acad Psychiatry*. 2024;48(1):52-56. doi:10.1007/s40596-023-01805-6

78. Owais S, Saperson K, Levinson AJ, et al. Evaluation of the online component of a blended learning electroconvulsive therapy curriculum for psychiatry residents to treat depression in older adults. *Acad Psychiatry*. 2024;48(1):36-40. doi:10.1007/s40596-023-01825-2

79. de Cates AN, Mullin D, Stirland L, da Costa MP, Tracy D. Breaking down barriers: promoting journals beyond the page with open access journal clubs. *BJPsych Bull*. Published online 2024. doi:10.1192/bjb.2024.3

80. Ben Ammer A, Bryan JL, Asghar-Ali AA. The Impact of COVID-19 in Reshaping Graduate Medical Education: Harnessing Hybrid Learning and Virtual Training. *Cureus*. 2024;16(3):e56790. doi:10.7759/cureus.56790

81. Brown TR, Amir H, Hirsch D, Jansen MO. Designing a Novel Digitally Delivered Antiracism Intervention for Mental Health Clinicians: Exploratory Analysis of Acceptability. *JMIR Hum factors*. 2024;11:e52561. doi:10.2196/52561

82. Noori S, Khasnavis S, DeCroce-Movson E, Blay-Tofey M, Vitiello E. A Curriculum on Digital Psychiatry for a US-Based Psychiatry Residency Training Program: Pilot Implementation Study. *JMIR Form Res*. 2024;8:e41573. doi:10.2196/41573
